# Increasing power for detecting awareness: a new approach to test group level objective performance

**DOI:** 10.1093/nc/niag039

**Published:** 2026-07-28

**Authors:** Shaked Lublinsky, Itay Yaron, William Marshall, Liad Mudrik

**Affiliations:** Sagol School of Neuroscience, Tel Aviv University, Tel Aviv, Israel; Sagol School of Neuroscience, Tel Aviv University, Tel Aviv, Israel; Department of Mathematics and Statistics, Brock University, St. Catharines, ON, Canada; Sagol School of Neuroscience, Tel Aviv University, Tel Aviv, Israel; School of Psychological Sciences, Tel Aviv University, Tel Aviv, Israel; Canadian Institute for Advanced Research (CIFAR), Brain, Mind, and Consciousness Program, Toronto, ON, Canada

**Keywords:** unconscious processing, consciousness, discrimination task, detection task, chance level performance, objective measure of awareness

## Abstract

Demonstrating reliable unconscious effects is notoriously challenging, and the scope of unconscious processing remains debated. Establishing unconscious effects requires two critical pieces of evidence: first, that the stimulus of interest was processed, and second, that participants were not consciously aware of that stimulus. Here, we focus on the second requirement which has faced criticism due to the low statistical power of awareness tests, undermining the reliability of evidence for unawareness. Consequently, reported unconscious effects might in fact stem from conscious processing. To better detect awareness, we suggest using two tests: the frequentist Group Binomial or Chi test and the Bayesian Group Binomial Bayesian test. Both tests were tailored for maximizing power in detecting consciousness, when using objective measures of awareness. We used simulations to compare the sensitivity and specificity of these tests with other commonly used ones (*t-*test, Mixed Model Logistic Regression, and Bayesian *t-*test). Our results show a clear power advantage for the proposed tests, across different scenarios. Furthermore, we reanalyzed 79 previously reported effects, from 15 papers on unconscious processing, and found cases where the proposed test revealed affects that were not detected by the other tests. We suggest that our systematic approach towards assessing and comparing the power of awareness tests will advance the field of unconscious processing and reduce the risk of contaminating reports of unconscious effects by undetected conscious processes.

## Introduction

Which functions depend on conscious processing, and which can operate unconsciously? This question has typically been studied by probing a specific function or process, and testing if it can take place even for stimuli that are not consciously perceived (Mudrik and Deouell 2022). The ongoing investigation of the depth and scope of unconscious processing has yielded a rich body of findings; some focus on lower-level effects like motor planning (Vorberg et al. 2003), color priming (Breitmeyer et al. 2004), or face orientation (Stein and Peelen 2021). Others targeted higher-level complex ones, such as action inhibition (van Gaal et al. 2009), semantic processing (for review see Kouider and Dehaene 2007), and task application (Van Opstal et al. 2010).

Critically, most of these findings were obtained using variants of the dissociation paradigm (or ‘simple dissociation’; Schmidt and Vorberg 2006). There, unconscious processing effects are inferred from two results: finding an effect on a measure of stimulus processing of the suppressed stimulus (most commonly relying on behavior or neural activity; henceforth, ‘indirect measure’), and then validating that participants are indeed unaware of that stimulus ‘via direct measures’. To illustrate, imagine an experiment where a masked prime stimulus precedes some visible target. Immediately thereafter, participants are asked to form some judgement on the target. The indirect measure here could be reaction times to the target. Then, a significant difference in the indirect measure between trials where the prime and the targets were related, and trials where they were not, can serve as indication that the prime was processed. Next, participants are asked to subjectively rate the visibility of the prime stimulus (subjective measure; Sandberg et al. 2010, Seth et al. 2008) and/or make some judgment about it, like they have done for the target (objective measure; e.g. report the presence/absence of the stimulus in a detection task or discriminating a feature of the stimulus among two possible choices; Reingold and Merikle 1988, Seth et al. 2008). These are the direct measures of awareness. The subjective measure should substantiate that participants did not perceive the stimulus based on their report. The objective one is used to quantify consciously available information about the prime based on participants’ performance. Specifically, by comparing their responses against the external stimulus or feature presented in each trial, one can estimate participants’ sensitivity to the prime (or to the feature of interest). Chance performance, or lack of sensitivity, supports the lack of awareness. Here, we focus on the objective measure.

To make the case that participants are indeed unaware using the objective measure, researchers are basically seeking to confirm the null. This can be criticized in two ways. One refers to the common use of Null-Hypothesis Significance Testing (NHST), which is not suitable for such a hypothesis, as NHST testing is focused on rejecting the null, while the purpose here is, in fact, to accept it (Dienes 2021, Stein et al. 2024). This is a valid point, which we will address later on. Here, we rather focus on the second line of criticism, which has been discussed, yet to a lesser extent; that in order for a null result to be meaningful, the test must be powerful enough to detect a genuine effect, if such an effect exists. Applied to studies of unconscious processing, validating the unawareness of the sample based on a lack of evidence for conscious processing might result in falsely inferring unconscious effects due to a lack of power to detect residual consciousness. Thus, and counter to the intuition about the power of statistical tests, in this case, low-powered awareness tests may actually drive ‘false-positive’ reports of unconscious effects.

Thus far, this point has mostly been discussed in the context of small sample sizes, rendering the awareness test underpowered (Shanks 2017, Vadillo et al. 2020, Meyen et al. 2022, Yaron et al. 2024). Yet, power also depends on the statistical procedure used to test a hypothesis, with some procedures being more powerful than others. In the case of unconscious processing studies, the hypothesis is that participants are not aware of the stimuli, typically assessed using performance on a forced-choice discrimination task. We argue that the commonly used tests (e.g*. t-*tests, Mixed Model Logistic Regression (MMLR)) do not maximize power when testing this hypothesis, and hold that it is crucial for the field to use the most powerful tests to validate the unawareness of participants. Importantly, this problem cannot be solved by merely using Bayesian statistics, since, as we show below, it applies to that as well.

### Why are current awareness tests underpowered?

Our criticism against the usage of common tests is driven by theoretical and practical reasons: First, these tests were devised to test a hypothesis about the ‘population average’, aiming at generalizing from the tested sample to the population. Yet in the unique case of unconscious processing studies, the goal is somewhat different. Here, the hypothesis actually focuses on the tested sample: we want to show that the specific participants we sampled are unaware of the stimuli, as a precondition for testing if they show an unconscious effect. Thus, we should demonstrate that the sampled group itself is at chance, and ideally, that this is true for every participant, and not only for the group-level average effect. If this is indeed the case, and if evidence for processing is found despite lack of awareness, we can then generalize our conclusion to the population and say that the tested process can take place without awareness.

Second, focusing on ‘group averages’ neglects the possibility that true group average of chance-level performance is concomitant with some participants having true above-chance performance that is being averaged out by other participants having below chance performance; at the extreme, even if half the participants are genuinely aware of the stimuli and have above-chance performance, the test will deem the group as being at chance if the other half show below chance performance (for discussions for how group level null effects might be explained by individual differences in opposing directions, see Ince et al. 2021, Yaron et al. 2025).

Lastly, testing hypotheses about the ‘population average’ requires estimating the variability of awareness scores in the population, at the cost of losing a degree of freedom, thereby somewhat reducing the test’s power. However, when testing for awareness in this context, there is no need to estimate this variability: under the null hypothesis that participants are truly unaware, performance in a forced-choice discrimination task should be fully determined by the properties of binomial distributions (the number of correct responses of each participant), taking into account the number of trials of each participant and the probability for generating a correct response by chance.

Accordingly, a more theoretically suitable test for awareness should be formulated on the ‘sample’, and rely on a more informed null hypothesis incorporating the information about the expected variability of participant-level awareness scores and not only about the group mean. We expect such tests to have more power, and to be robust to outlier participants (specifically high-performing, hence conscious, participants), which is known to reduce the power of *t-*tests (Zimmerman 1994).

### The proposed approach

We suggest here a frequentist test and an additional Bayesian solution that overcome the above-mentioned issues. Researchers can thus choose their preferred test, taking into account the objection to using a frequentist approach of confirming the null for showing performance is not above chance (Stein et al. 2024). Codes for these tests can be found in our repository: https://github.com/ShakedLub/GroupLevelObjectivePerformance.

The **frequentist test** combines two tests, which complement each other and provide high power. The first is the ‘group binomial (GB) test’, a Z-test based on the asymptotic normality of the binomial distribution (with 20 trials or more, convergence towards normality is commonly considered sufficient; otherwise an analytic solution or bootstrapping-based solution can be safely used; see Supplementary Chapter 6 for an implementation of the latter). That is, the null hypothesis is that all participants are at chance. This is formulated in the test in the following manner: the average awareness scores are distributed according to a normal distribution, $N\left(\mu = chance,\sigma ={\sigma}_s\right)$, where ${\sigma}_s$ is fully determined by sample size and chance-level performance ($chance=0.5$ for two alternatives discrimination tasks). Hence, GB tests for above-chance group-level awareness while considering the expected variability of average awareness scores with chance-level performance. This test focuses on the sample rather than on generalizing to the population and does not require estimating the variability.

However, the GB test is based on the group mean, and is insensitive to samples comprised of mixtures of true below and above-chance performance. We complement the GB test with the Chi-squared (${\chi}^2$) goodness of fit test, which is sensitive to any deviance from chance-level performance (either below or above chance). Although the Chi-squared test solves all problems we highlighted above, it has a price (namely, reduced power compared to the GB test) when all aware participants differ from chance in the same direction (i.e. having true awareness scores consistently above or below chance level performance). Thus, we recommend a combined test that takes both the results of the Chi-squared and GB tests into account.

To summarize, the combination of the two methods is aimed at providing a sufficiently powered frequentist test for the purpose of testing awareness in studies of unconscious processing. In a nutshell, each test compensates for the ‘blind spot’ of the other test: the GB test has high power when all participants deviate from chance in the same direction (e.g*.* all are above chance) and low power when participants deviate from chance in opposite directions relative to chance (i.e. some are above chance and some below). The Chi-squared test has higher power than GB in the latter case, but lower in the former (see Supplementay Chapter 1 and Supplementary Fig. 1).

The **Bayesian solution** relies on the Bayes Factors approach (Dienes 2014) to compare a model of conscious processing with a model of unconscious processing. To that end, we define models that follow the same logic underlying the frequentist tests described above. Specifically, the unawareness model was implemented as a simple binomial model predicting chance-level performance for all participants. Then, the alternative model assumes that the sample may include both aware participants, whose performance is determined according to a hierarchical model, and unaware participants, for which performance is at chance, using the same binomial approach (see the Materials and methods section for details about the model specification and prior settings). We term the Bayes Factor weighting evidence in favor of each model the ‘Group Binomial Bayesian test’ (henceforth ‘GB-Bayes’).

### Comparing the sensitivity of common methods versus the proposed approach

To compare the power of the proposed solution with that of existing tests (namely, *t-*test, MMLR, and the Bayesian *t-*test; see Supplementary Chapter 3 and Supplementary Fig. 3 for an additional analysis), we used systematic simulations. In the simulations, we examined four different scenarios (for more details see the Methods section): (i) all participants are truly unaware, (ii) most participants are truly unaware, while a quarter of the full sample is truly aware, (iii) the true average awareness score is at chance, but all the true awareness scores deviate from chance, being either below or above chance with equal probability, and (iv) all participants are aware with true awareness scores distributed narrowly around a close-to-chance awareness score. These simulations allowed us to understand the pros and cons of each test, comparing the power and false-positive rates across tests and scenarios, and having full control of the ground truth regarding the presence and degree of awareness in each sample.

In addition to the simulation study, we reanalyzed existing datasets, applying all tests on 79 different tested effects from 15 studies examining unconscious processing (See Supplementary Chapter 7 for details about each study). By combining the results of both simulations and real data we demonstrated the practical benefit of replacing the commonly used tests with the suggested ones, which are arguably more sensitive for detecting awareness.

## Materials and methods

### Simulations

All simulations we conducted focused on objective awareness scores, defined as accuracy in a forced-choice discrimination task with two alternatives. We examined how well different statistical tests capture above-chance performance when it exists and when it is absent. Also, we manipulated both the number of trials (${n}_i$ = 100, 200 or 300) and the number of participants (i.e*.* sample size; $K$ = 10, 20, 30 or 50), to examine their effect on the performance of the different tests. Each simulation included 10 000 iterations, in which the performance on a forced-choice discrimination task is simulated for each participant $i$, from a binomial distribution with two parameters: ${\pi}_i$= true awareness score, and ${n}_i$= number of trials.

More formally, we define ${y}_{ij}$ as the outcome of the ${j}^{th}$ trial of the ${i}^{th}$ participant. If ${\pi}_i$ is the participant’s true awareness score, then ${y}_{ij}=1$ with probability ${\pi}_i$. Given ${n}_i$ representing the number of trials of the ${i}^{th}$ participant, then:


(1) \begin{eqnarray*} {x}_i={\sum}_{j=1}^{n_i}{y}_{ij}\sim Binomial\left({n}_i,{\pi}_i\right), \end{eqnarray*}

where ${x}_i$ is the total number of ‘successes’ in ${n}_i$ trials and ${p}_i=\frac{x_i}{n_i}$ represents the estimated awareness score for this participant.

We examined four different cases: one in which all participants are unaware; a mixed case, where some participants are aware and some are not; and additional two cases where all of them are aware. The percentage of aware participants in each case was accordingly defined as $D$, and set to 25%, 100% and 100%, respectively. The true awareness score of unaware participants was fixed at 0.5 (chance level performance according to a forced-choice discrimination task with two alternatives) For each aware participant the true awareness score was sampled from a truncated normal distribution with ($\mu$, $\sigma$) and distribution limits between ($a,1)$ ($\sim TN\left(\mu, \sigma, a,1\right)$ where $a$ denotes the lower truncation bound and 1 is a fixed upper bound; see Table 1 for the parameter values in different simulations). The code used to conduct these simulations is available at https://github.com/ShakedLub/GroupLevelObjectivePerformance.

**Table 1:** Summary of simulation parameters.

	Unaware sample	Mixed sample	Aware sample with a small spread slightly above chance	Aware sample with a large spread around chance
Percent of aware participants ($D$)	0	25%	100%	100%
Average true awareness score of aware participants	-	0.564	0.532	0.5
Standard deviation of true awareness scores of aware participants	-	0.04	0.02	0.19
Minimal true awareness scores of aware participants ($a$)	-	0.5	0.5	0

In the unaware sample, true awareness scores of all participants were defined as 0.5.

To evaluate the performance of the proposed solutions in these cases, four simulations were conducted, one for each case. The first examined the ‘Unaware sample’ scenario, and focused on the false positive rate (FPR) of the different tests (requiring an FPR lower than $\alpha =0.05$). FPR was estimated as the proportion of iterations where the test deemed performance to be above chance out of all iterations. The next three simulations focused on the power of the tests in different scenarios. In all of them, power was estimated as the proportion of iterations where the test deemed performance to be above chance (the probability of correctly rejecting the null when it is false). In the ‘Mixed sample case’ (*D* = 25%), the true awareness scores were sampled from a truncated normal distribution with ($\sim TN\left(\mu =0.55,\ \sigma =0.05,\ a=0.5,1\right)$). This simulation was aimed at mimicking a situation where the true average awareness score is close but higher than chance (here, 0.516; here and below we report the group-level awareness scores and standard deviations after truncation) due to the sample including both aware and unaware participants. In the ‘Aware sample with a small spread slightly above chance’ simulation, we mimicked a scenario where the awareness scores are centered on a value that is slightly above chance. Specifically, true awareness scores were sampled from a truncated normal distribution with ($\sim TN (\mu =0.525,\ \sigma =0.025,\ a=0.5,1$)). The resulting true average score at the group level was 0.532. Conversely, in the ‘Aware sample with a large spread around chance’ simulation, awareness scores for all participants were sampled from a truncated normal distribution with (~$TN\left(\mu =0.5,\ \sigma =0.2,\ a=0,1\right)$)$.$ This mimics a situation where participants are either truly above or below chance (the latter case can happen, as can be seen in the empirical data we obtained; see Supplementary Fig. 8. A possible explanation for such a pattern is that participants might show sensitivity to the stimuli, but consistently mix up the button assignment when providing their response). Thus, when true awareness scores are averaged, the resulting value is near 0.5, although at the individual level, no true awareness score is 0.5.

In all simulations, our proposed solutions were tested against three commonly used tests, two frequentists (*t-*test, MMLR) and one Bayesian (Bayesian *t-*test; here using the R function ttestBF from the BayesFactor package (Morey et al. 2015), with the default prior settings). The frequentist tests were one sided, testing awareness scores against 0.5 (chance performance of a forced-choice discrimination task with two alternatives), using $\alpha =0.05$. The MMLR model included a fixed intercept for the sample, as well as a random effect intercept for each participant (in the common coding syntax: $accuracy\sim 1+\left(1| participant\right)$). The significance of the group-level intercept was tested using the Wald test to assess if performance was above chance, converting the *P*-value to be one sided. The MMLR was implemented in R using the glmer function from lme4 package. For Bayesian testing, two thresholds are typically used to interpret BFs: moderate evidence for ${H}_0$ is commonly inferred when $B{F}_{01}>3$, inconclusive evidence is inferred when $\frac{1}{3}<B{F}_{01}<3$, and moderate evidence for ${H}_1$ is inferred when $B{F}_{01}<\frac{1}{3}$. Here, for the purpose of assessing Bayesian tests in a more comparable manner to the frequentist tests, we took a liberal approach and defined the threshold for a positive result (supporting ${H}_1$) as evidence for conscious processing ($B{F}_{01}<\frac{1}{3}$). This is liberal, since even in cases where the test does not find conclusive evidence for the null, we will consider the results as suggesting that participants were not aware. For completeness, in Supplementary Chapter 2, we also report the results of the Bayesian tests when taking the evidence for the null as the critical threshold ($B{F}_{01}>3$).

**Figure 1:**
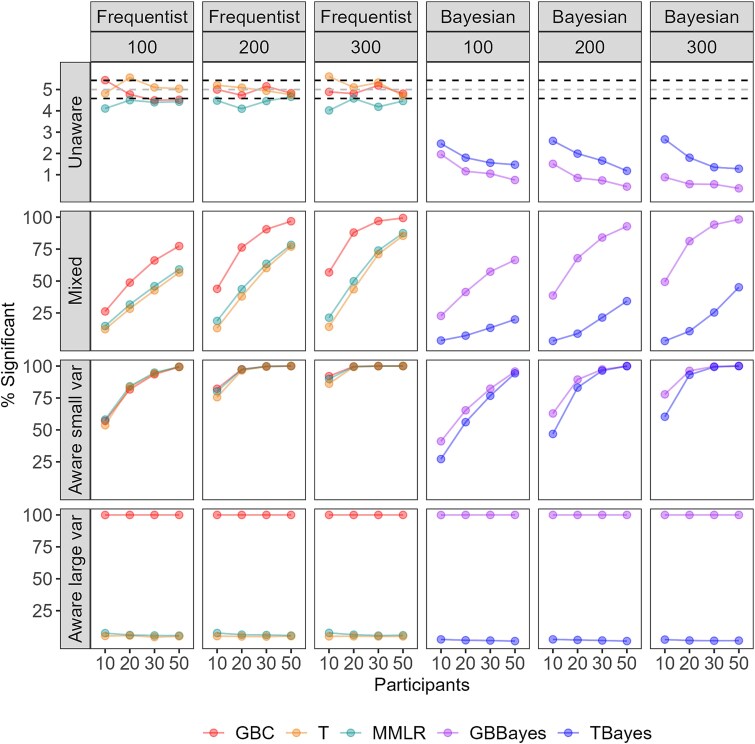
Simulations were used to compare the performance of five tests: GBC test, GB-Bayes test, t test, Bayesian *t-*test (TBayes) and MMLR. The x-axis denotes the number of participants in the sample. The y-axis indicates the proportion of significant iterations out of 10 000. Each row is a different simulation such that in the first row, the y-axis indicates the false positive rate (FPR), and in the following rows it represents power. The frequentist tests are presented in the left columns, where the red, orange, and green lines represent GBC, T and MMLR tests correspondingly. The Bayesian tests are presented in the right columns, in which the blue and purple represent TBayes and GBBayes, respectively. For both Bayesian tests evidence for ${H}_1$ is presented. The columns are the number of trials for each participant. In the first row the black horizontal dotted line represents the confidence interval around $\alpha =0.05$, that was calculated from a binomial distribution for this $\alpha$ based on the number of iterations (10, 000) and the gray horizontal dotted line marks 0.05.

However, unlike frequentist tests, for Bayesian ones the false-positive rate is not calibrated to maximize sensitivity while fixing false positive rate to a given $\alpha$ (see the upper panel of Fig. 1). Hence, to compare Bayesian and frequentist tests, we complement this analysis with a Receiver Operating Characteristic (ROC) one. We examined the performance of all tests across all possible significance thresholds to estimate the sensitivity of the tests to group-level awareness. The performance of the tests was quantified by the Area Under the Curve (AUC). Here, AUC indicates the classification performance of each test according to the p-values or BFs, separately for every simulated scenario, sample size and number of simulated trials. Specifically, the *P*-values or BFs obtained in each simulated iteration of the mixed, large spread and the small spread scenarios were treated as ‘signal’ cases, whereas those obtained in the unaware sample scenario were treated as ‘noise’ cases. These values were then submitted to ROC analysis using the ‘pROC’ R package (Robin et al. 2011).

### Proposed solutions

The frequentist version of our solution, the ‘GBC test’, combines the results of the ‘Group Binomial (GB) and Chi-squared test (${\mathrm{\chi}}^2$).’

#### The GB test

The GB tests whether the group level (average) performance is higher than chance. The test uses the asymptotic normality of binomial distributions for each individual’s awareness score, and hence the normality of the awareness score distribution across participants. It uses a Z-test, in which the expected variability in the null distribution is calculated according to the number of trials and participants.

Specifically, each ${p}_i$ is asymptotically normal, such that for large ${n}_i,{p}_i=\frac{x_i}{n_i}\sim N ({\pi}_i,{\sigma}_i^2 )$ where ${\sigma^2}_i= Var\ \left(\frac{x_i}{n_i}\right)=\frac{1}{n_i^2\ } Var\left({x}_i\right)=\frac{n_i{\pi}_i\left(1-{\pi}_i\right)}{{n_i}^2}=\frac{\pi_i\left(1-{\pi}_i\right)}{n_i}$, and since the null hypothesis is that all participants are unaware (${\pi}_i=0.5\forall i$) we can compute the variability directly without the need to estimate it from data.

Accordingly, to calculate a Z score we use the formula:


(2) \begin{eqnarray*} z=\frac{\overline{p}-\mu }{\frac{\sigma }{\sqrt{K}}}=\frac{\overline{p}-0.5}{\frac{1}{\sqrt{K}}\sqrt{\frac{\sum_{i=1}^K\frac{\pi_i\left(1-{\pi}_i\right)}{n_i}}{K}}}=\frac{\overline{p}-0.5}{\frac{1}{K}\sqrt{\sum_{i=1}^K\frac{0.{5}^2}{n_i}}} \end{eqnarray*}

#### Chi-squared test for goodness of fit

We used a variant of the Chi-squared test, based on the premise that it will be sensitive to detect deviations from chance performance irrespective of their direction. Also, here the number of degrees of freedom is K (as opposed to K-1 that is commonly used for the chi-squared test), because the hypothesis is more restrictive and does not require estimating the expected observations.

The statistic ${\chi}^2$ was calculated as:


(3) \begin{eqnarray*} {\chi^2}_{\left( df=K\right)}={\sum}_{i=1}^K\ \left(\frac{{\left.\left({x}_i-\frac{n_i}{2}\right.\right)}^2}{\frac{n_i}{2}}+\frac{{\left.\left(\left({n}_i-{x}_i\right)-\frac{n_i}{2}\right.\right)}^2}{\frac{n_i}{2}}\right) \end{eqnarray*}

Where ${x}_i$ is the number of correct (i.e. successful) trials, $\left({n}_i-{x}_i\right)$ is the number of incorrect (*i.e.* failed) trials, and $\frac{n_i}{2}$ is the expected number of both correct and incorrect trials (according to the probability of a correct trial under the null hypothesis (0.5 in a forced-choice discrimination task with two alternatives).

#### The group binomial or chi (GBC) test

As we show below, each one of these tests (GB and Chi-squared) is more powerful than the other under different circumstances. Thus, we combined them into one test that would be robust in all scenarios, such that a positive result in one of the tests is enough to deem performance as above chance. The *P*-value was defined as twice the minimal p-value between the GB and Chi-squared tests. Doubling the *P*-value is done to avoid FPR inflation due to performing two tests, and is applied as an adjustment for the combined GBC test *P*-value while keeping $\alpha =0.05$ (analogous to a Bonferroni correction).

#### The group binomial Bayesian (GB-Bayes) test

To provide a Bayesian alternative to the GBC test, we relied on Bayesian Factors (BF; Dienes 2014). Specifically, GB-Bayes compares the evidence for a null model assuming that all participants are unaware (${M}_{H0}$), with the evidence for a hierarchical mixture model that allows for aware participants (${M}_{H1}$). Thus, ${M}_{H1}$ includes two subpopulations: aware and unaware participants. The resulting BF can then be used to quantify evidence in favor of unawareness versus awareness at the group level.

In either model, the number of correct responses of each participant is modeled as ${x}_i\sim Binomial\left({\pi}_i,{n}_i\right)$. For ${M}_{H0}$, it is assumed that all participants are unaware, and thus ${\pi}_i=0.5$. By contrast, ${M}_{H1}$ defines an additional parameter ${\delta}_i$ which determines whether each participant belongs to an aware sub-population (${\delta}_i=1$) or an unaware subpopulation (${\delta}_i=0$). The ${\delta}_i$’s are given uninformative priors (${\delta}_i\sim Bernoulli(0.5)$), so that the model does not make any *a-priori* assumptions about the prevalence of aware participants. For participants belonging to the unaware sub-population (${\delta}_i=0$) the true awareness score is set to chance (${\pi}_i=0.5$), while for participants belonging to the aware sub-population the prior distributions of the awareness scores are normally distributed (${\pi}_i\sim N\left(\theta, {\sigma}^2\right)$); where $\theta$and $\sigma$ denote the true average awareness score and standard deviation of true awareness scores, respectively (with priors settings of $\theta \sim TN\left(\mu =0.55,\sigma =0.1,a=0.5,1\right)$ and $\sigma \sim TN\left(\mu =0.025,\sigma =0.05,a=0,1\right)$ for all analyses). The choice of priors aligns with the most important case—when the average awareness scores are tightly grouped just above chance. If the priors are misspecified and higher awareness scores are more frequent, then all the proposed methods have sufficient power, and optimal prior selection is less important. In Supplementary Chapter 4, we present the results of the same model with uninformative (Supplementary Fig. 4) and alternative plausible priors on $\theta$ and $\sigma$ (Supplementary Fig. 5).

To compare the two models, we created a ‘supermodel’ including both ${M}_{H0}$ and ${M}_{H1}$, using a binary indicator variable ($M$) to control whether ${M}_{H0}\ \left(M=0\right)$ or ${M}_{H1}\ \left(M=1\right)$ is used, with an uninformative prior ($M\sim Bernoulli(0.5) )$. The posterior probabilities for each model given the observed data, $P\left(M=0|x\right)$ and $P\left(M=1|x\right)$, were estimated using a Monte Carlo Markov Chain procedure, using two chains with a burning period of 1500 and additional 5000 samples per chain. Then, we defined the Bayes factor $B{F}_{01}$ as $\frac{\ P\left(M=0|x\right)\ }{P\left(M=1|x\right)\ }$, the ratio between the posterior probabilities of ${M}_{H0}$ and ${M}_{H1}$ (Lodewyckx et al. 2011, Lindeløv 2018).

### Datasets

Datasets were collected from 15 previously reported studies of unconscious processing resulting in 97 tested effects (see a summary in Supplementary Table 1). We strived to obtain as many datasets as possible, as long as they measured objective awareness using forced-choice discrimination with two alternatives. To that end, we first used the studies that were analyzed in Yaron et al. 2025 and Yaron et al. 2024, and then added one more study (Van Opstal and Rooyakkers 2022), following correspondence with the author. Datasets were collected by contacting the first author of the paper, and using open-access datasets published online.

Four additional publications used in those papers were excluded: in two publications the objective awareness task was not a forced-choice discrimination task with two alternatives, one paper did not have an objective awareness measure, and in another paper, the objective measure results were not provided.

Out of the included 97 effects, 18 were further excluded: four had no objective awareness measure, six tested conscious effects (in studies which examined both conscious and unconscious processes), in two the objective awareness task was not a 2AFC task (in this case, only the specific effects which did not use a forced-choice discrimination task with two alternatives were omitted, while keeping the tested effects which did use such a task), three tested effects used the breaking Continuous Flash Suppression procedure (and accordingly their results might not reflect unconscious processing; Stein et al. 2011), in one the number of trials as reported in the manuscript did not result in integers when calculating correct trials from the success rate, and for another tested effect, a mismatch was found with respect to the number of participants, such that a different number of participants were reported compared to the number of participants in the database. Finally, for one tested effect the data from the objective measure for aware participants was missing. The resulting datasets thus included 79 tested effects.

### Analysis

All datasets were analyzed using the above-mentioned tests: *t-*test, MMLR, Bayesian *t-*test, GBC, and GB-Bayes (for the Bayesian tests, using the same priors as in the simulations). Importantly, in all these analyses we did not exclude any participant based on objective performance due to concerns of regression to the mean (Shanks 2017), even when such exclusion was done in the original study (this was the case for 18 tested effects; note that we did exclude participants due to other reasons, in line with the original works). To complement the analyses reported here, we also repeated them while using only the participants who were included in the original studies, using a resampling approach (see **Resampling with Criterion test** and **Absolute Value Resampling with Criterion test** in Supplementary Chapter 6). Notably, we did not correct *P*-values for multiple comparisons in this analysis since our purpose was not to reinterpret the findings of any specific paper, but rather to compare the outcomes of the different tests on the same empirical data.

## Results

### Simulations

#### Specificity

By and large, the false positive rates of all frequentists tests were not > $0.05$ in the ‘Unaware sample’ simulation where all samples included only truly unaware participants (Fig. 1, first  row). This reassures us that the tests are unlikely to yield a positive result in the absence of an effect. Similarly, for both Bayesian tests we found evidence for ${H}_1$ in <5% of iterations in all conditions.

#### Sensitivity

**Figure 2:**
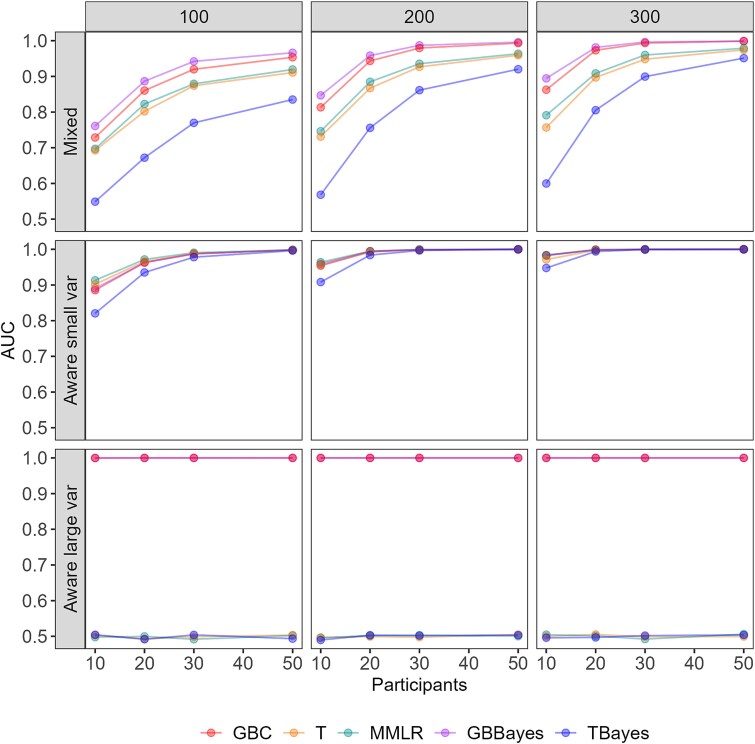
Comparing the area under the curve (AUC; given on the y-axis) of all tests. Each point denotes the AUC in a given condition (*i.e.* specific number of trials and participants), computed following an ROC analysis. Conventions are the same as in Fig. 1.

Here, we move to scenarios where the sample is mixed or even fully aware, and ask how sensitive the tests are. In the ‘Mixed sample’ simulation, where 25% of the participants are aware with an average true awareness score of 0.564, the GBC was most powerful among the frequentist tests, reaching close to 100% power with 200/300 trials and 50 participants. Similarly, the GB-Bayes test was most sensitive among the Bayesian ones (lowest and highest difference between GB-Bayes and Bayesian *t-*test [19%, 71%]) (Fig. 1, second  row). When comparing between frequentist and Bayesian tests, the ROC analysis shows that GBC and GB-Bayes have the highest AUCs, showing sensitivity to awareness irrespective of a specific threshold (Fig. 2, first  row).

In the ‘Aware sample with a small spread slightly above chance’ simulation, where all participants are aware with an average true score of 0.53, comparable results were found across frequentist tests, while the GB-Bayes test still performed a bit better than the Bayesian *t-*test (Fig. 1, third  row). A similar trend was observed in the ROC analysis, with a minor exception showing a small advantage for MMLR in conditions of low number of participants and trials (Fig. 2, second  row).

Importantly, in the ‘Aware sample with a large spread around chance’ simulation, where the average performance is still at chance yet the individual scores substantially deviate from chance, the superiority of the GBC and GB-Bayes tests was most pronounced, as they showed close to 100% sensitivity across all numbers of trials and participants, while the other tests were close to 0%. This was also the case when comparing the sensitivities of the tests under the ROC analysis (Fig. 2, last  row).

### Datasets re-analysis

**Figure 3:**
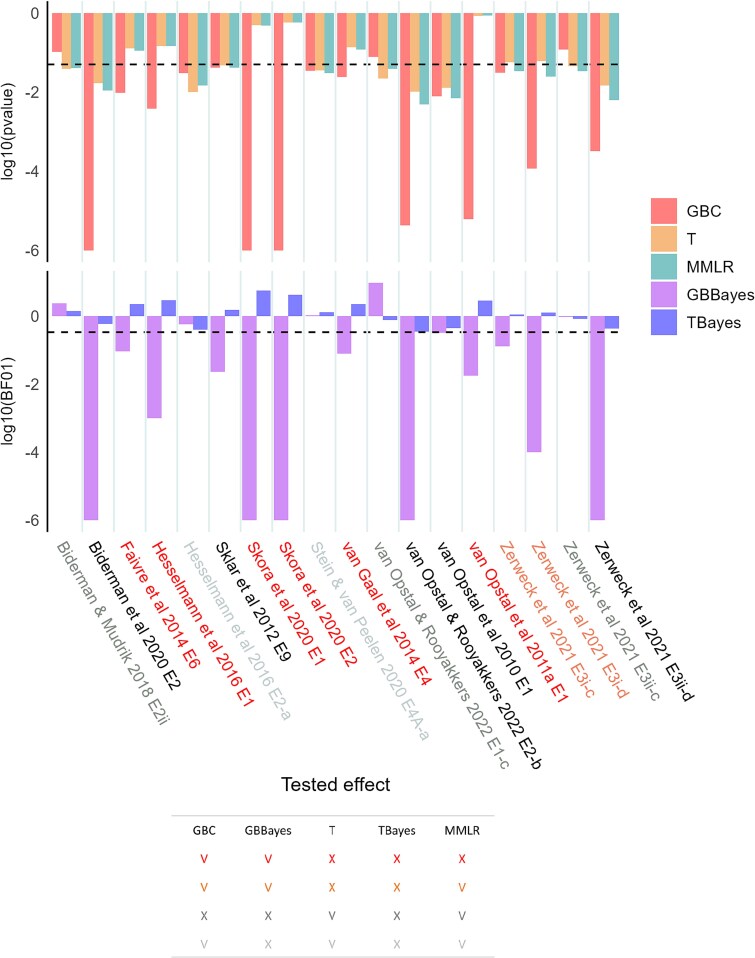
Datasets re-analysis: Only tested effects in which there was a disagreement between the 5 tests (GBC test, GB-Bayes test, t-test, Bayesian *t-*test and MMLR) on the significance of the group are presented (N = 18; in the remaining 61 tested effects, all tests found significant or nonsignificant results). The upper plot presents the frequentist tests and the lower plot the Bayesian tests. The y-axis is log10(p-value) in the upper plot and log10($B{F}_{01}$) in the lower plot, the x-axis denotes the tested effect’s label (where ‘E’ is an abbreviation for experiment, and the number specifies which experiment it is within the manuscript. A capital letter is added (*e.g.* 3A) if the authors named this experiment with a number and letter in the original manuscript. To refer to the specific condition a letter separated by a hyphen is used (*e.g.* ‘-a’). Roman numerals (*e.g.*‘i’,‘ii’) represent the measure of awareness. Publications from the same author and the same year are differentiated using the letters a/b. In the upper plot, the horizontal black line denotes the significance threshold of all frequentist tests ($\alpha =.05$; notably, due to correction for multiple comparisons, the GBC *P*-values are twice the minimum p-value of its two constituent tests). In the lower plot, the horizontal black line indicates the threshold of Bayesian tests for showing evidence for ${H}_1$ ($B{F}_{01}$=⅓), (evidence for ${H}_0$ is presented for both the Bayesian tests, so that evidence for ${H}_1$ will be downwards, same directionality as the frequentist plots). P-values and BFs below ${10}^{-6}$ were modified to this value. Each color depicts the p-value of a different test (same colors as in Fig. 1). To present the comparison between the GBC, GB-Bayes and the commonly used tests in the field, differential results between the tests are marked with color (see table): Red marks tested effects in which the GBC and GB-Bayes tests are significant and T, MMLR and Bayesian *t-*test are not. Orange marks tested effects in which the GBC, GB-Bayes and MMLR tests are significant, and T and Bayesian t-tests are not. Dark gray filling marks tested effects that are not significant according to GBC and GB-Bayes (and also the Bayesian t-test) but are significant according to T and MMLR. Light gray marks tested effects where GB-Bayes and Bayesian *t-*test did not find evidence supporting the M1 model but are significant according to GBC, T and MMLR.

We analyzed the 79 tested effects to compare the performance of GBC and GB-Bayes tests with the other commonly used tests. The results were largely compatible with the simulation conclusions: For 61 of the tested effects, the results of all tests agreed. From the remaining 18 tested effects, six indicated above-chance performance only according to the GBC and GB-Bayes tests, and in two other tested effects, this was true also for the MMLR which concurred with the GBC and GB-Bayes positive result. On the other hand, the GBC and GB-Bayes tests (and also Bayesian *t-*test) didn’t detect effects in three tested effects that were found significant by *t-*test and MMLR. Finally, in two tested effects the two Bayesian tests—GB-Bayes and the Bayesian *t-*test—did not suggest above-chance performance, while all frequentist tests did. When comparing the Bayesian tests, GB-Bayes found evidence for ${H}_1$ in 13 out of the 18 tested effects (with mismatches between the tests), whereas this was not the case in any tested effects for Bayesian *t-*test (Fig. 3). Supplementary Chapter 5 provides more details about the distributions and results of the datasets: The distributions of awareness scores in the tested datasets where all tests agreed are presented in Supplementary Figs. 6 and 7. Datasets in which a disagreement between the tests was found are presented in Supplementary Fig. 8, and for these cases, Supplementary Figs. 9 and 10 present the p-values of frequentist tests and the BFs of Bayesian tests, respectively.

Overall, we interpret these results as strengthening our suggestion that the GBC test is the most sensitive of the frequentist tests, and the GB-Bayes test is the most sensitive of the Bayesian ones.

## Discussion

When studying unconscious processes, a major challenge is to make sure that participants are indeed unaware of the presented stimuli. Here, we suggest a novel approach for doing so, relying on two group level awareness tests: the GBC frequentist test and the GB Bayes, Bayesian one. Both ask if *all* participants are unaware, as opposed to examining group-level awareness. We used simulations to compare our tests with commonly used ones (*t-*test, MMLR, Bayesian *t-*test), and showed that our tests outperform the others in four scenarios. We further applied all tests on experimental data from 15 studies examining 79 effects of unconscious processing and corroborated the simulation results.

Our tests were found to have more power than the commonly used tests in all the simulated scenarios in which there were conscious participants, including the situation where the sample contains a small proportion of participants with slight above-chance performance. Thus, these tests are better able to detect awareness in the most critical cases, in which commonly used tests will often erroneously deem the sample as unaware. Such erroneous conclusions can potentially drive false-positive reports of unconscious effects that are actually driven by undetected conscious participants (Shanks 2017, Meyen et al. 2022). This power advantage stems from theoretical and statistical reasons. First, as opposed to common tests, our tests were tailored to answer the specific question of interest; whether all participants in this specific sample are indeed unaware. This is different from asking the more general question about the population average that is usually tested. One implication of this change in question is that one no longer needs to estimate the variability of awareness scores in the population, thereby losing a degree of freedom, which reduces the test’s power. Instead, the variability under the null can be fully calculated from the sample size and number of trials per participant. Second, focusing on the group average inevitably leads to a failure to detect potential cases where high awareness scores (i.e. conscious participants) cancel out with low awareness scores (i.e. substantially below chance). However, any deviation from chance may be indicative for awareness. Hence, the group average might not be sensitive enough to detect awareness in such cases.

Importantly, the proposed tests are primarily designed to investigate unconscious processing effects within the widely used ‘simple dissociation’ paradigm (Schmidt and Vorberg 2006, Schmidt and Biafora 2023). Under this paradigm, unconscious effects are demonstrated by establishing both the presence of a processing effect and the complete unawareness of the relevant content (note that the latter requirement is not needed under the ‘double dissociation’ and ‘sensitivity dissociation’ paradigms; Meyen et al. 2022, Schmidt and Vorberg 2006). Our tests specifically focus on demonstrating complete unawareness. That is, convincingly demonstrating that participants were unaware of the presented stimuli. While doing so is notoriously challenging (Meyen et al. 2022, Rothkirch et al. 2022, Stein et al. 2024), our tests are able to mitigate some of this challenge, by enhancing the sensitivity of awareness tests.

Another means we took to increase the meaningfulness of the test is to complement the NHST approach with a Bayesian one, in line with previous suggestions in the field of unconscious processing (Rouder et al. 2007, Dienes 2014, 2015, 2021). Specifically, BFs allow researchers to obtain evidence for unawareness by evaluating support for a null awareness model in comparison to an alternative model that incorporates awareness, based on experimental data and specified priors (e.g. Goldstein et al. 2022, Stein et al. 2024; Notably, equivalence tests, which were not examined here, provide a frequentist approach towards the same goal, Lakens et al. 2018). Such model comparison provides flexibility in tailoring priors to fit the requirements of unconscious processing studies (e.g. defining half-normal priors on awareness scores to reflect the hypothesis that they are above chance; Skora et al. 2023). Notably, our results showed that Bayesian solutions (including ours) are not immune to errors: both tests suggested substantial evidence for unawareness in over 20% of several conditions under the Mixed simulated scenario where 25% of the sample were aware of the stimuli (see Supplementary Fig. 2; *cf*. Appendix F in Meyen et al. 2022). Yet despite this issue, the overall performance of the Bayesian tests was satisfactory. Importantly, as long as the sample size is large enough (here, > 20), the GB-Bayes test was less prone to errors than the Bayesian *t-*test (using a default prior; note that this was also found, though to a lesser extent, when the GB-Bayes test was also run with weakly informed priors). We argue that akin to the GBC test, this advantage stems from the way the test was defined: the GB-Bayes test aims to find evidence favoring a null model which assigns null awareness to all participants, as opposed to existing Bayesian solutions that again focus on the group level or test each participant individually.

Beyond the tests we introduce here, another aspect of our work focuses on the ability to assess the power of the test used to examine participants’ awareness. Previous work has already shown that awareness tests tend to be underpowered, focusing mostly on the number of trials needed to provide sufficient power (Shanks 2017, Vadillo et al. 2022, Yaron et al. 2024). Here, we suggest that it might be informative to also assess and report the power of the statistical test itself, by measuring it under simulated scenarios held by the researchers to likely represent the obtained data. In the current work, we explored critical cases where we want our tests to detect conscious contamination, and examined how our tests fair in each of these scenarios. We suggest that taking a similar approach when conducting empirical work can enhance the reliability of future studies: if researchers define in advance the expected patterns they would like to test (*e.g.* the expected percentage of conscious participants, the expected range of their responses), they could test *a-priori* the power of their objective measure to detect such cases, and report their results (the code we provide here can be easily modified to accommodate other scenarios of interest). This would allow the community to judge the reliability of the claims for unconscious processing.

Three potential concerns about our work should be taken into account. First, our tests were all measured against scenarios that did not involve participant exclusions. Since this is a relatively common practice in the field, one might question the relevance of our results to studies that follow it. Typically, such exclusions are done to make sure that all included participants are indeed unaware of the stimuli. However, we opted not to simulate them, given that they introduce potential risks due to post-hoc selection of extreme cases and Regression to the Mean (Shanks 2017, Stein et al. 2024, Yaron et al. 2024). To ensure our results are not confined to scenarios where no participants are excluded, we tested our approach also when post-hoc selection is implemented (see Supplementary Chapter 6 and Fig.  11). Again, our tests outperformed the others.

Second, we did not include all possible scenarios in our simulations, and our re-analysis of the datasets does not necessarily represent the entire field (as these datasets were not collected in a systematic manner, akin to a meta-analysis search). We acknowledge this pitfall, though we argue that the scenarios we chose do represent plausible cases that are relevant to the question at hand. We encourage future studies to complement these scenarios with other ones. As for the datasets, they were collected by searching for relevant work (i.e. studies of unconscious processing that used an objective measure) and approaching as many researchers of the detected papers as we could. To the best of our knowledge, this constitutes the largest re-analysis effort in the field thus far (N tested effects = 79). However, we used it solely for the purpose of evaluating the different tests, rather than drawing conclusions on the findings themselves, given that they were not systematically collected. We welcome further attempts to collect more datasets and reanalyze them, preferably in a systematic, meta-analytic manner, to complement the findings reported here.

Finally, we note that our Bayesian tests were conducted while using informative priors for the GB-Bayes test and the default prior for the Bayesian *t-*test (and the priors can heavily affect the results; see Kass and Raftery 1995. For a study that examined the effects of priors on Bayesian analysis of the objective measure, see Stein et al. 2024). The chosen prior setting targets a scenario for which it is arguably difficult to detect an effect, because only a proportion of participants have slightly above chance performance. Thus, even if this prior is misspecified (*e.g.* the true prior holds that there are participants with awareness levels substantially above chance) it would make it easier for the test to detect an effect, such that the impact of misspecification will be minor. In short, the chosen prior prioritizes the lowest power situations, representing the worst-case scenario for detecting an effect. Regardless, the choice of prior could potentially explain the advantage of the GB-Bayes test, especially given that its priors and model structure resemble the simulated variables and the data structure in the scenarios we examined. To test if this can indeed account for the GB-Bayes test advantage, we also evaluated its performance with uninformative and alternative plausible priors (see Supplementary Chapter 4). Though this has indeed lowered the GB-Bayes test performance, its advantage compared with the Bayesian *t-*test was largely maintained. Hence, while we recommend the GB-Bayes test due to its superior performance, we also acknowledge potential avenues for improvement, particularly in prior specification and in the computational procedures used to estimate BFs, which can affect their robustness and reliability (*e.g.* see Schad et al. 2021).

## Conclusions

A prerequisite for demonstrating unconscious processing is providing robust evidence for participants’ lack of awareness of the stimulus of interest. In light of prior criticisms regarding contamination by conscious processing, we propose here two tests designed to maximize sensitivity to awareness and demonstrate their power advantage through simulations and reanalysis of empirical datasets. Additionally, we highlight the importance of incorporating power analysis into awareness testing. We provide a conceptual framework for doing so, as well as codes that would ease the implementation of such analysis in future work, and the development of new tests incorporating our conceptual contribution regarding the null hypothesis in awareness testing. Together, these contributions advance the methodological rigor of unconscious processing research and help mitigate the risk of misattributing conscious effects to unconscious phenomena.

## Supplementary Material

Supplementary_material_niag039

## Data Availability

All data and codes used in this project are publicly available on our open access repository: https://github.com/ShakedLub/GroupLevelObjectivePerformance. In the root folder of the repository, you can find documented implementations of the GBC (in R and MATLAB) and GB-Bayes (in R only) tests. To run the simulations and reanalyze the datasets, please refer carefully to the README.md file.
