# Supplementary material for: Increasing power for detecting awareness: a new approach to test group level objective performance
Source: Neurosci Conscious. 2026 Jul 28;2026(1):niag039. doi: 10.1093/nc/niag039 (PMC13411290; doi:10.1093/nc/niag039)
Supplement: Supplementary_material_niag039 [file supplementary_material_niag039.zip › GBC_and_GB_Bayes_Supplementary_Material_niag039.docx]

**Supplementary Material:** **Increasing power for detecting awareness: A new approach to test group level objective performance**

The supplementary material presents additional analyses that complement those reported in the main manuscript. In the first chapter, we demonstrate why we decided to combine the GB and Chi-squared tests into the GBC one, by directly comparing these three tests. In the second chapter, we present the results for the Bayesian tests (including for the $H_{0}$ model). In the third chapter, we compare the performance of an alternative test, the global null test, to the ones we propose. In the fourth chapter, we use uninformed priors. In the fifth chapter, the awareness score distributions of all tested effects are presented, while indicating which tests suggested above-chance performance. In the sixth chapter, we repeat the analysis of the tested effects in the obtained datasets when applying participant exclusion. Lastly, in the seventh chapter, we describe and characterize all reported studies used in the analysis.

**Chapter 1: Simulations comparing GBC, GB and Chi-squared tests**

The same simulations wereused as in the main analysis, yet here, comparing the GBC, GB and Chi-squared tests. In the *Unaware sample* simulation, all tests generally showed the expected specificity, with most values within the confidence interval of $\alpha=0.05$ (Supplementary Figure 1). In the *Mixed sample* simulation, all three tests had high performance, which was overall comparable, with a slight advantage for the GBC test. In the *Aware sample with a small spread slightly above chance* simulation, the Chi-squared test had considerably lower power than GB and GBC tests. Finally, in the *Aware sample with a large spread around chance* simulation, the GB test failed to detect an effect in 63% of the iterations on average across conditions, while the other two tests were at 100% detection rate. Taken together, these simulations demonstrate that both the Chi-squared and the GB test are sometimes complementary; when one test fails, the other succeeds. Thus, combining them together allows us to preserve their sensitivity while not lowering their specificity.


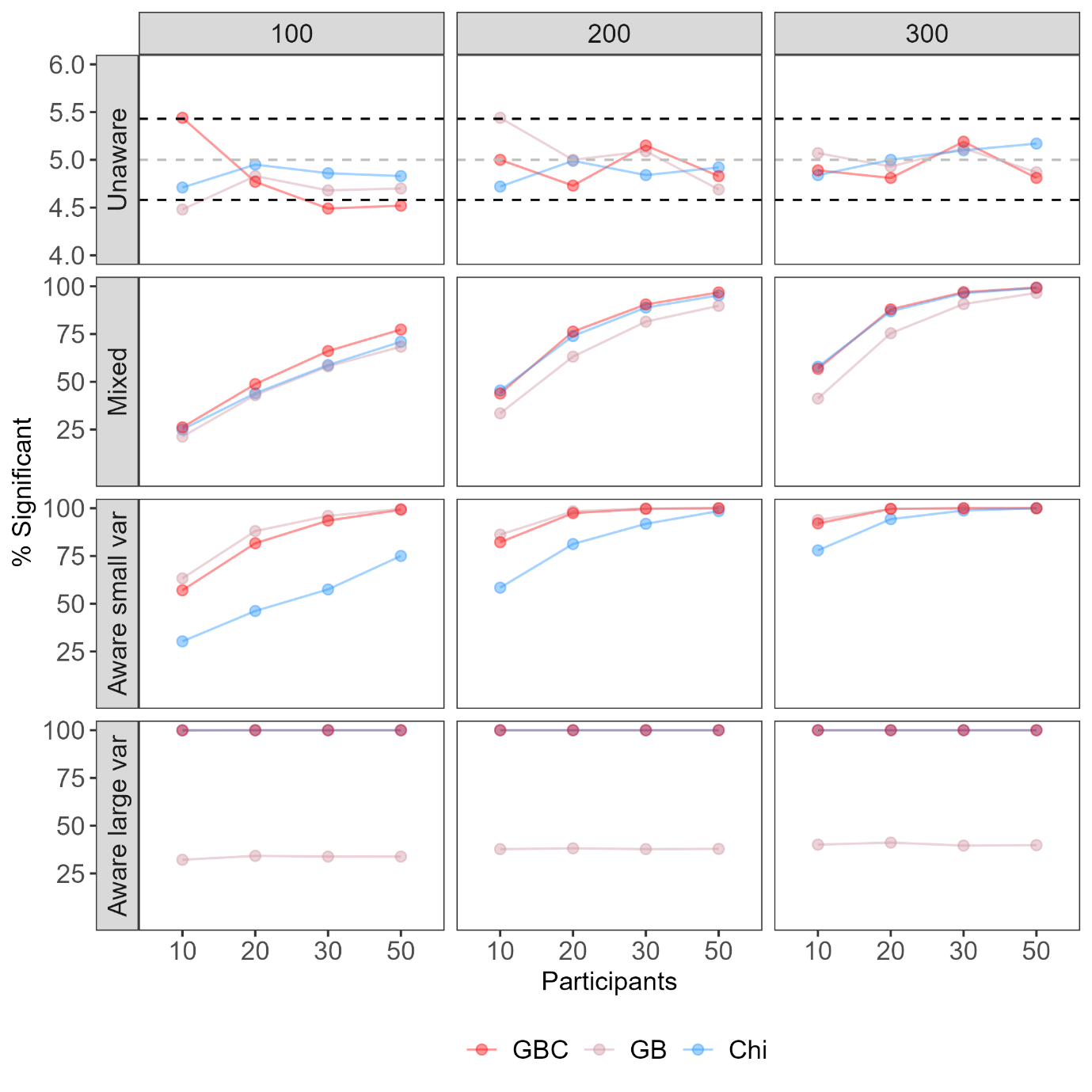


**Supplementary Figure 1:** Performance comparison of the GBC (red), GB (pink) and Chi-squared (dodger blue) tests. Conventions are the same as in Figure 1.

**Chapter 2: Simulations comparing Bayesian tests on evidence for the null**

The results of the Bayesian tests in favor for the null (i.e., for unawareness) were not presented in the main text, as the focus there was on comparing the new suggested tests (GBC and GB-Byes) to the commonly used ones (t-test, MMLR, Bayesian t-test), while using the same terms in the frequentist and Bayesian tests for comparability. Here, for completeness, we also present the results when focusing on the evidence for the null as the critical threshold, by defining the threshold as $BF_{01}>3$.

- - 1. **Specificity**

Interestingly, with this threshold, the GB-Bayes was more sensitive than the Bayesian t-test, irrespective of the number of trials and participants. For example, for 200 trials and 30 participants GB-Bayes found evidence for the null in 23% more iterations than the Bayesian t-test. This again suggests that the Bayesian t-test might be less suited for testing objective awareness than GB-Bayes.

- - 1. **Sensitivity**

In the *Mixed sample* simulation, the Bayesian t-test only provided evidence for awareness when the number of participants and trials was high. For example, in a sample of 30 participants with 100 trials each, the Bayesian t-test found evidence for the null in 30% of the iterations, despite the presence of truly aware participants in the sample. This may indicate that the Bayesian t-test is less suited for this scenario, which is arguably plausible (Supplementary Figure 2, second row). In the *Aware sample with a small spread around chance* simulation, the results of the two tests are quite similar. Importantly, in the *Aware sample with a large spread around chance* simulation, where the true average performance is at chance, yet the individual participants' true scores substantially deviate from chance, the Bayesian t-test found evidence for the null in the vast majority of the iterations when simulating samples with more than 20 participants, even though in this scenario the sample includes only aware participants (Supplementary Figure 2, fourth row).


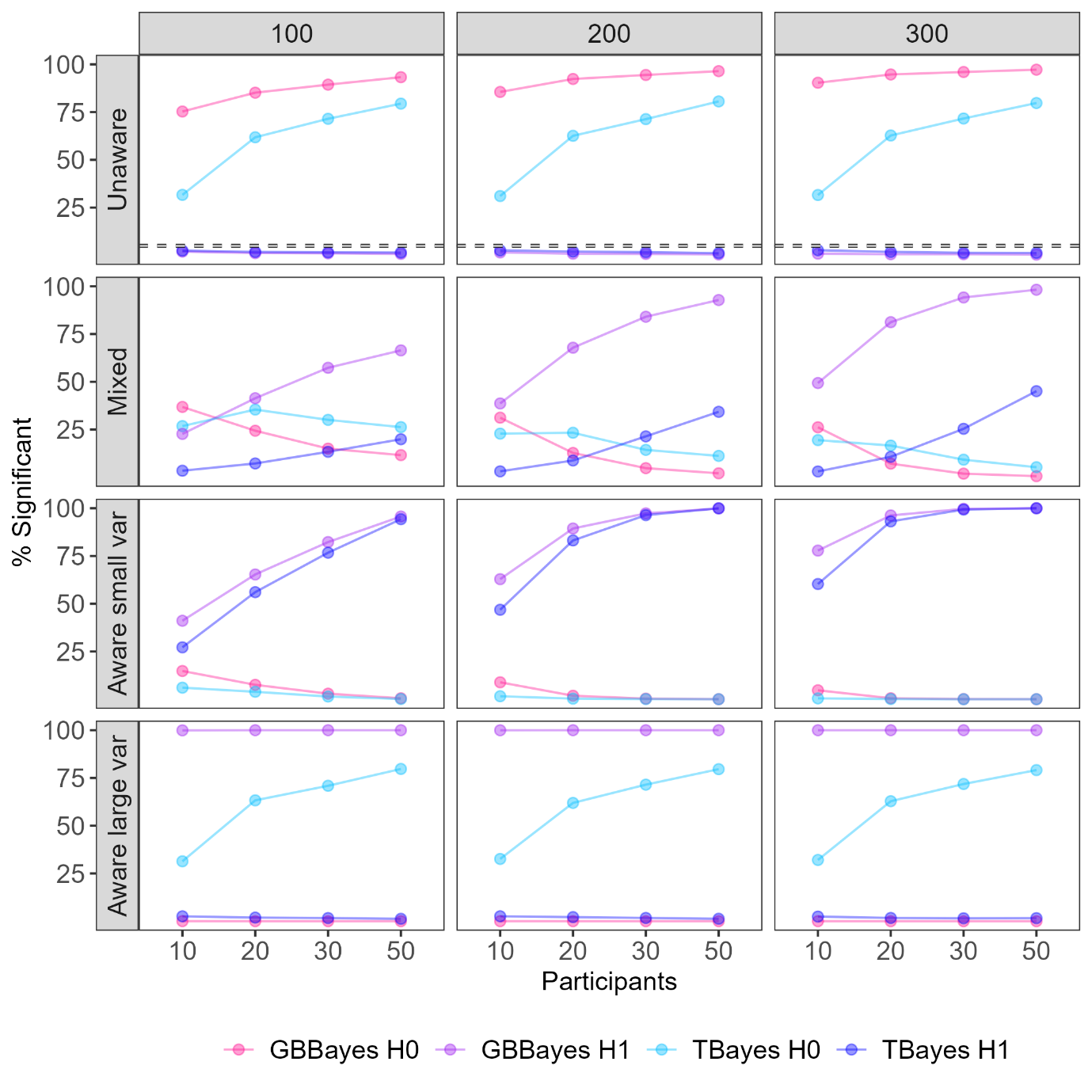


**Supplementary Figure 2:** Performance comparison of the GBBayes $H_{1}$ and $H_{0}$ (purple and pink, correspondingly), and Bayesian t-test $H_{1}$ and $H_{0}$ (blue and light blue, correspondingly) tests. Conventions are the same as in Figure 1.

**Chapter 3: Comparing test performance with the global null test**

An alternative test for the hypothesis that all participants are unaware, is to test for awareness in every single participant. A candidate test implementing this idea is the *global null* test (Donhauser et al., 2018). The global null test first examines the significance of individual-level effects, and then tests if the number of significant effects is larger than expected given the $\alpha$ rate of the individual-level tests. Here, we used two-sided binomial tests to examine individual-level awareness ($\alpha=0.05)$, and then another binomial test to examine whether the proportion of individual-level effects is larger than the expected proportion of significant effects if all participants are unaware (i.e., the false-positive rate of the individual-level tests^[[1]](#footnote-1)^). By comparing the results obtained by the global null test with the GBC, we can see that GBC has higher power in all conditions (expect for the large spread scenario, where both tests perform similarly well), in parallel to the expected false-positive rate being under 5% (similar results were found following an ROC analysis; see Supplementary Figure 3).

**
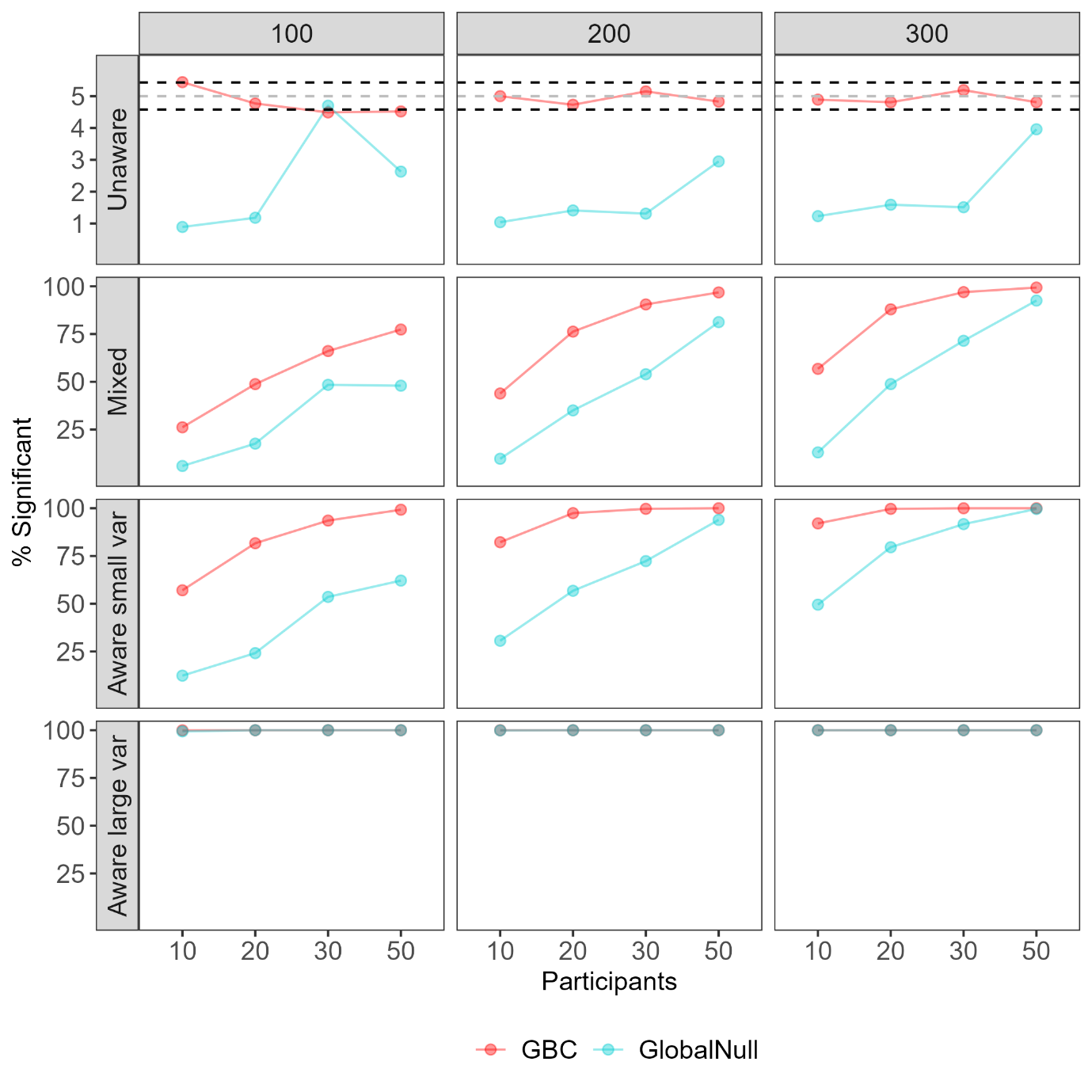
**

**
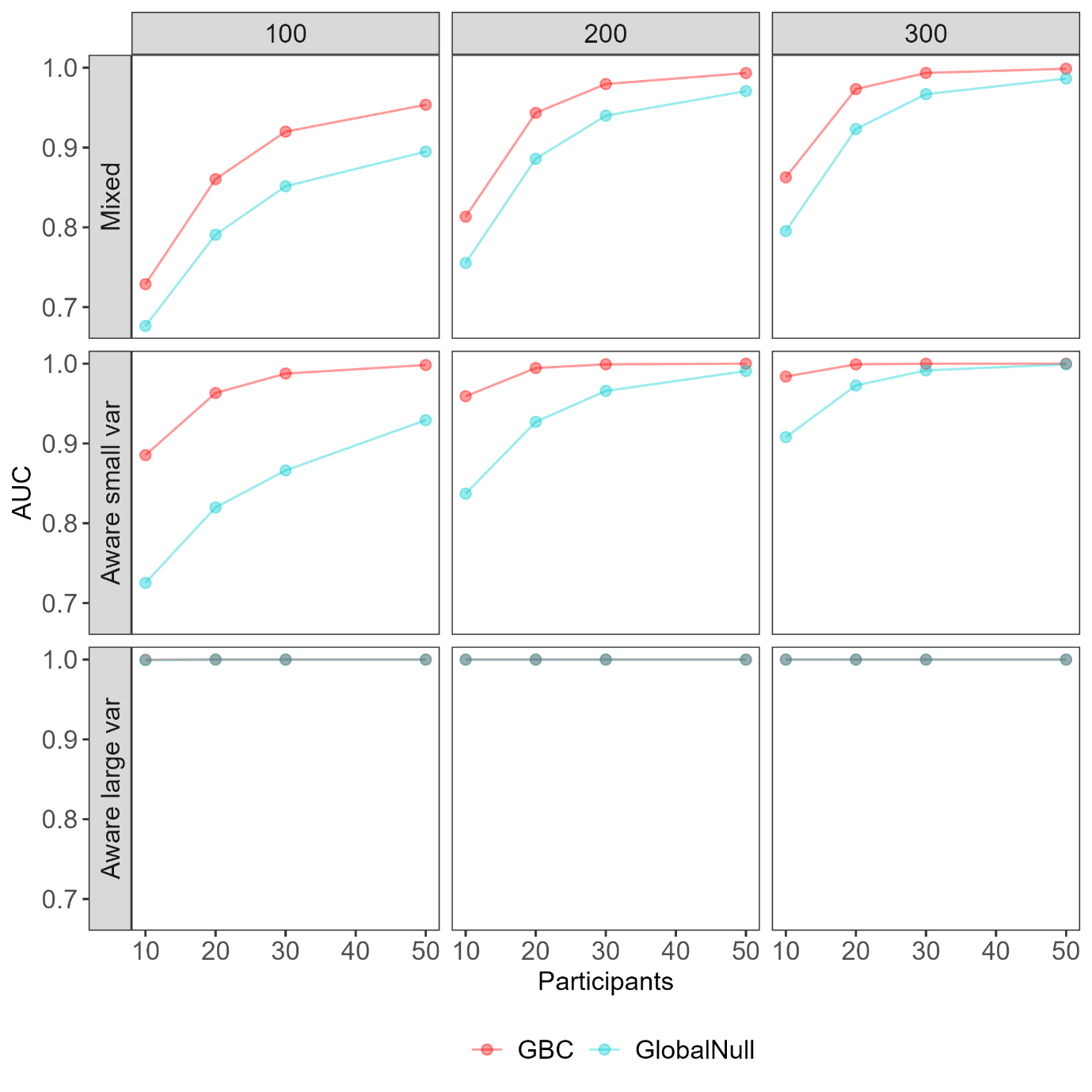
**

**Supplementary Figure 3:** Comparing the performance of the *global null* and GBC tests. Cyan lines denote the performance of the *global null* test. Upper panel: Comparing the percent of significant results for the two tests according to a single threshold ($p< 0.05)$. Lower panel: Assessing the performance of the tests according to the AUC measure, following ROC analysis (see Methods section in the main text). Same conventions are used as in Figure 1 and Figure 2 for the upper and lower panels, respectively.

**Chapter 4: GB-Bayes performance with uninformative and alternative priors**

To examine to what extent the performance of the GB-Bayes test is driven by the informative priors used in the main text and generally assess prior sensitivity, we analyzed its performance while using either uninformative priors or alternative plausible prior settings. Specifically, the prior on both the average ($\theta$) and standard deviation ($\sigma$) of the group of aware participants was manipulated accordingly: First, in the uninformative priors analysis, we used uniform priors ($\theta\sim U\left( 0, 1 \right)$ and $\sigma\sim U (0, 0.5)$, respectively). Thus, a-priori, all possible values of both parameters were equally probable^^[[2]](#footnote-2)^^. Second, we used three different parametrizations of the priors for each parameter. Specifically, we manipulated the $\mu$ and $\sigma$ parameters of the truncated normal distributions for $\theta$and $\sigma$ such that we simulated the GBBayes sets with: “Wide $\sigma$” ($\mu= 0.55$, $\sigma=0.15$ for the $\theta$parameter and $\mu= 0.025$, $\sigma=0.1$ for the $\sigma$ parameter), “High $\mu$“ ($\mu= 0.6$, $\sigma=0.1$ and $\mu= 0.05$, $\sigma=0.05$), “Both” ($\mu= 0.6$, $\sigma=0.15$ and $\mu= 0.05$, $\sigma=0.1$) prior settings. Notably, we used longer chains and burning periods in both scenarios (set to 15,000 and 30,000 respectively in the uninformative priors analysis and to 3,000 and 10,000 in the alternative priors analysis) to allow the MCMC procedure to converge, and 1,000 iterations were used in each simulated condition (rather than the 10,000 iterations used in all other analyses) in the latter analysis to reduce simulation runtime.

The results show clearly that the original model outperforms the alternative models. First, the uninformed model showed lower performance in almost all conditions, with a main pitfall of finding evidence for the null in a very high percent of the simulation iterations when the sample size and the number of trials are both small. However, overall the results of the uninformative model still outperform the Bayesian t-test according to an ROC analysis (lower panel of Supplementary Figure 4), considering all possible thresholds, and when sample size and number of trials are high. Second, comparing model performance across different alternative weakly informed prior settings yielded qualitatively similar patterns compared with the Bayesian t-test (see Supplementary Figure 5), albeit as could be expected, with a smaller power advantage. Again, a relatively higher portion of iterations showed evidence for the null in the aware sample scenarios (especially when manipulating the $\sigma$ parameter, representing individual differences in the awareness of conscious participants; see the lower panel of Supplementary Figure 5), which declined for large sample sizes. Yet, overall, the model’s behavior under the different prior settings remained largely similar, suggesting a relative robustness to prior selection.

**
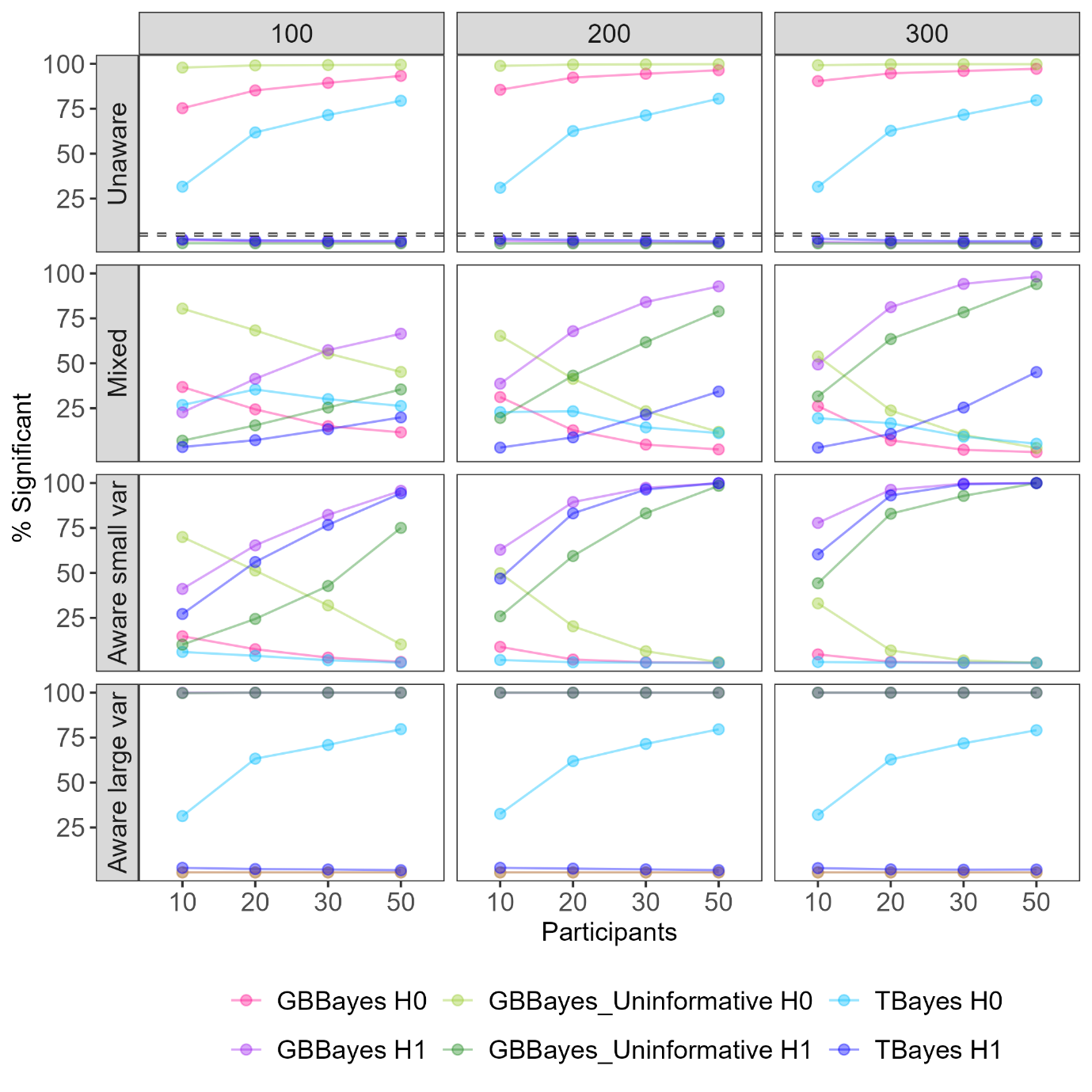
**

**
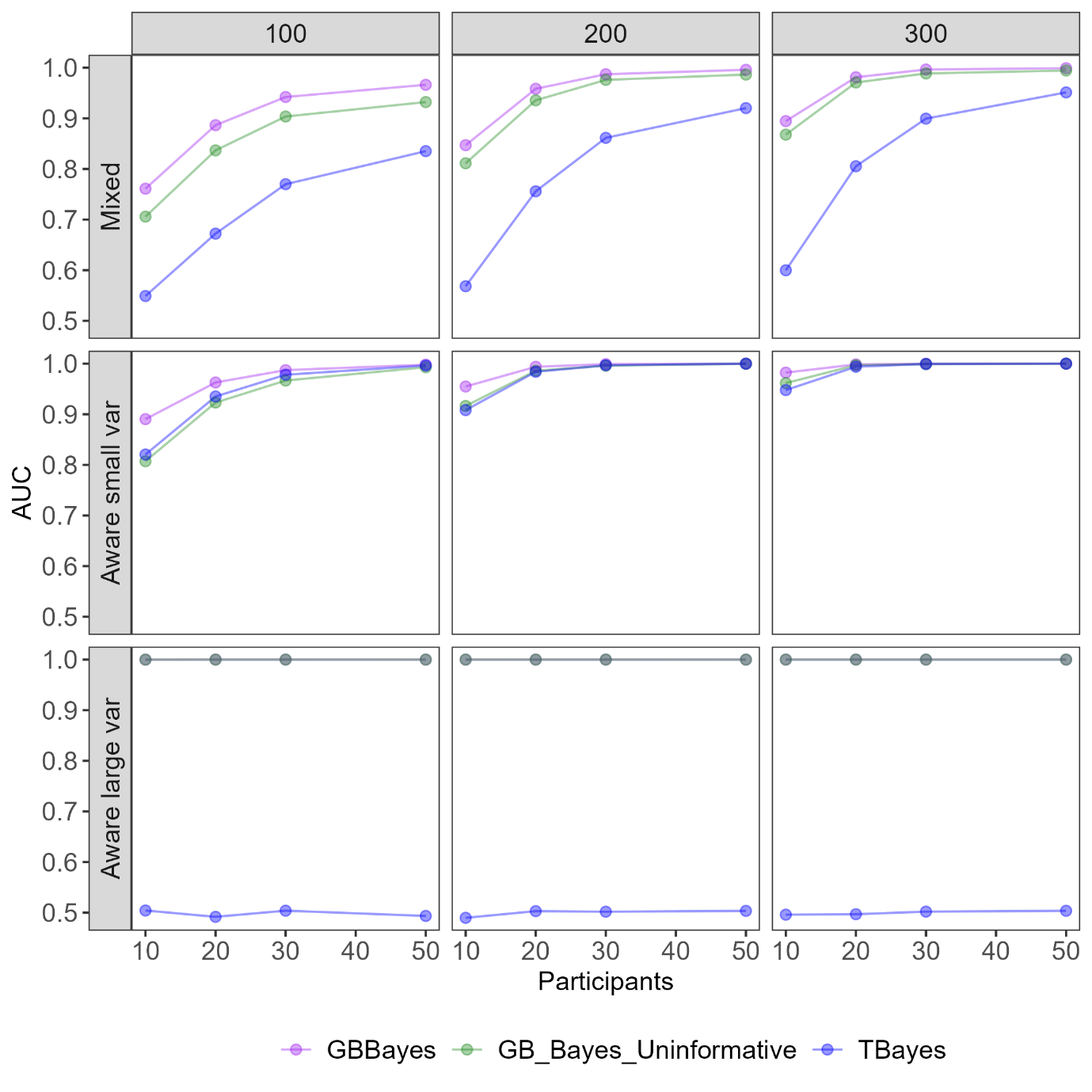
**

**Supplementary Figure 4:** Performance comparison of the GBBayes test with informative priors (purple and pink lines for $H_{1}$ and $H_{0}$, respectively), the GBBayes test with uninformative priors (dark and light green lines for $H_{1}$ and $H_{0}$, respectively), and the Bayesian T-test (TBayes; blue and light blue lines for $H_{1}$ and $H_{0}$, respectively). Upper panel: Comparing the percent of iterations where evidence for $H_{1}$ or $H_{0}$ was obtained by each test according to a single threshold ($BF_{10}> 3)$. Lower panel: Assessing the performance of the tests according to the AUC measure, following ROC analysis. Conventions are the same as in Supplementary Figure 3.

$\theta$ prior sensitivity:


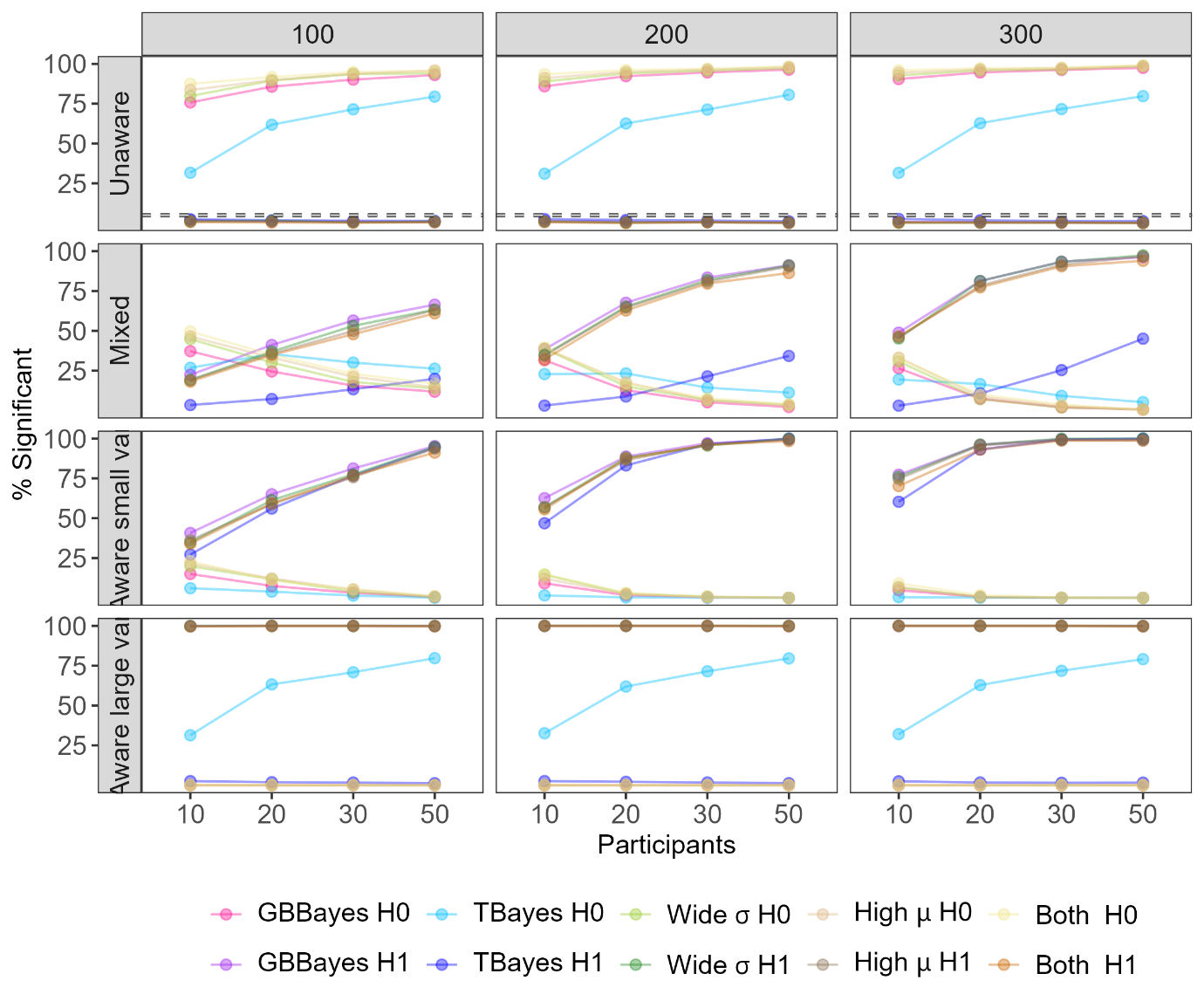


$\sigma$ prior sensitivity:


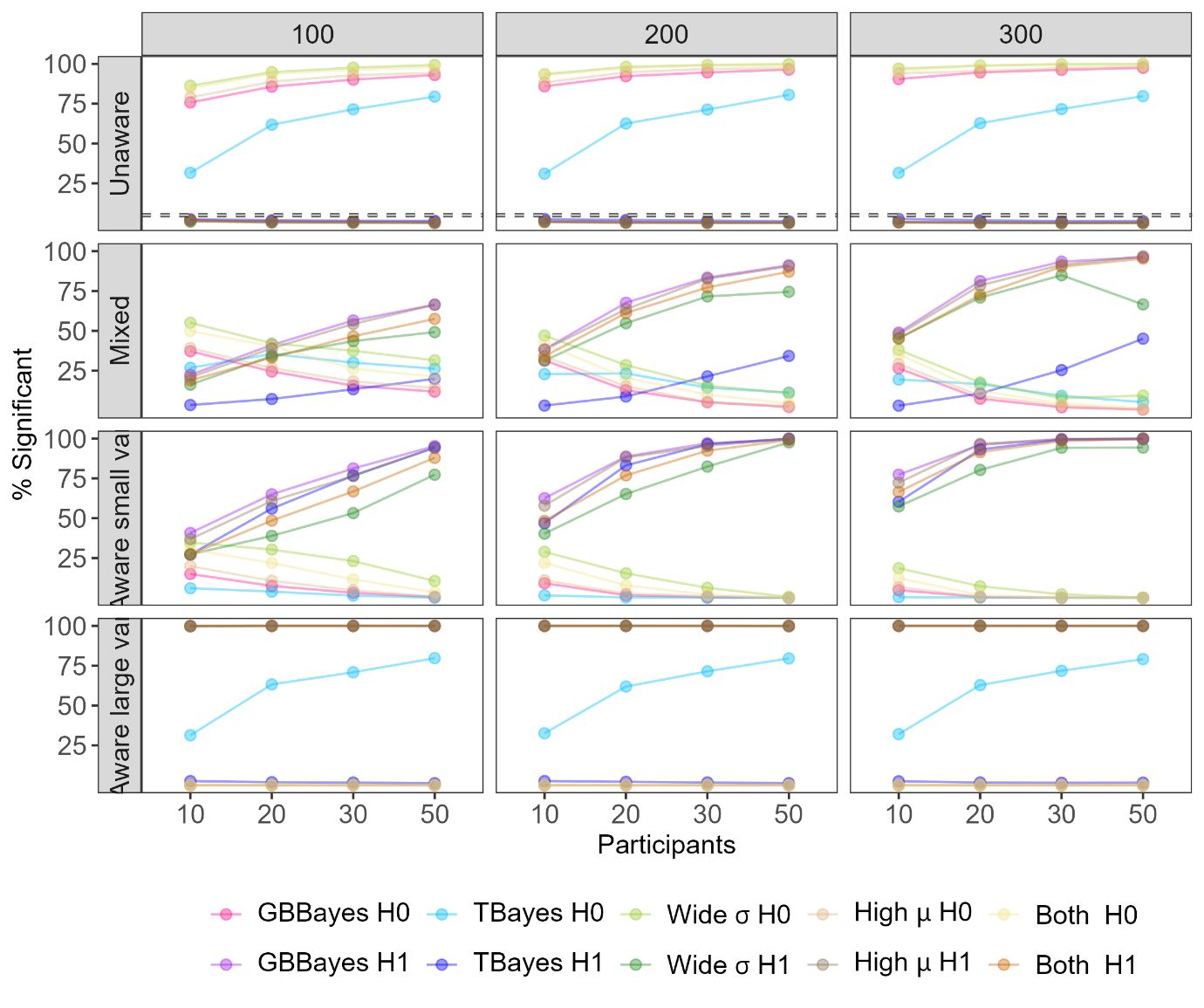


**Supplementary Figure** 5**:** Performance comparison of the GBBayes test with informative priors (purple and pink lines for $H_{1}$ and $H_{0}$, respectively), the Bayesian T-test (TBayes; blue and light blue lines for $H_{1}$ and $H_{0}$, respectively), and the GBBayes test with uninformative priors in three different prior settings for the $\mu$ and $\sigma$ truncated normal distribution parameters of the $\theta($upper panel) and $\sigma$ (lower panel) model parameters: Wide $\sigma$ (dark and light green lines for $H_{1}$ and $H_{0}$, respectively), High $\mu$ (dark and light brown lines for $H_{1}$ and $H_{0}$, respectively), and Both (indicating using both wide $\sigma$ and high $\mu$; dark and light orange lines for $H_{1}$ and $H_{0}$, respectively). Conventions are the same as in Supplementary Figure 3.

**Chapter 5: Distribution of awareness scores for the tested effects analyzed in the main manuscript**

To better characterize the distribution of awareness scores for the different tested effects we analyzed, we present histograms of the data. Supplementary Figures 6 and 7 depict performance histograms of tested effects for which all tests agreed that performance is above chance or not, respectively. Supplementary Figure 8 presents performance histograms of tested effects for which a disagreement between tests was found. For these tested effects, Supplementary Figures 9 and 10 present the p-values of frequentist tests and the BFs of Bayesian tests, correspondingly.

**
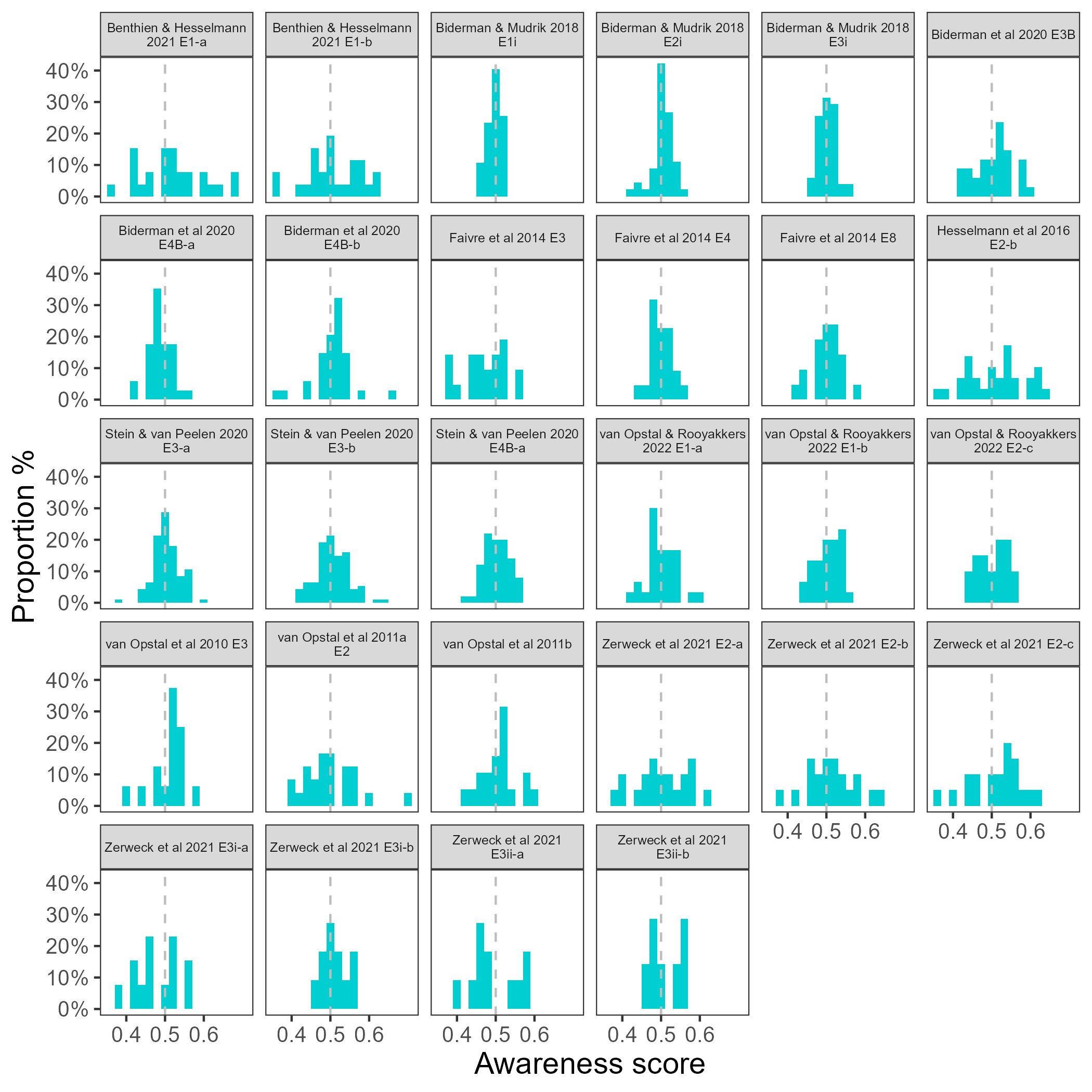
**

**Supplementary Figure 6:** Awareness scores histograms where all tests suggested performance not to be above chance. The tested effect name is written in the title. Y axis indicates the proportion of participants in each bin and x axis denotes the awareness scores, with all subplots using the same scale (ranging between 0.3 and 0.7). The dashed gray line indicates chance level performance (0.5).

**
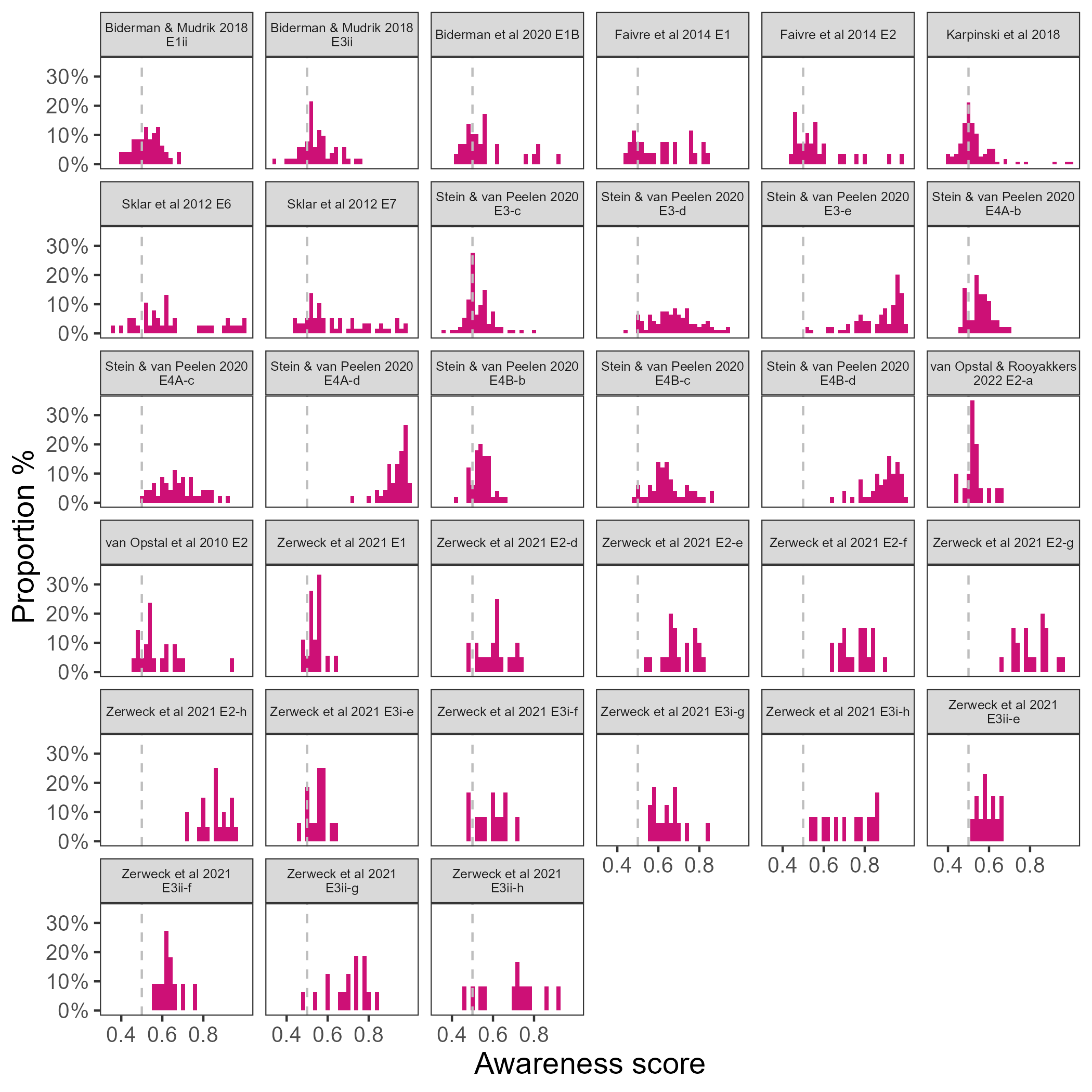
**

**Supplementary Figure 7:** Awareness scores histograms where all tests suggested performance to be above chance. Figure conventions are the same as in Supplementary figure 6, yet here the X-axis scale ranges between 0.3 and 1.

**
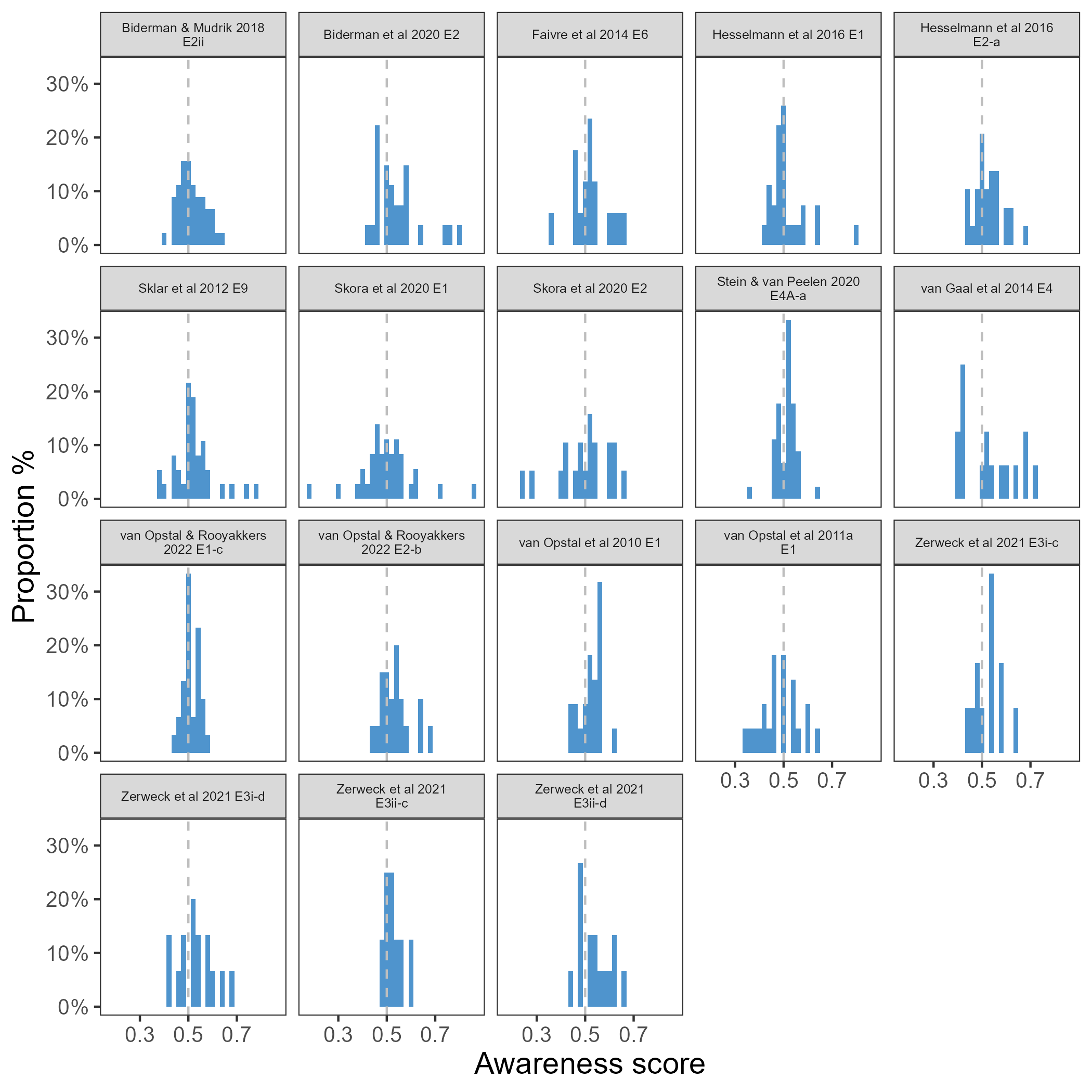
**

**Supplementary Figure 8:** Awareness scores histograms where the different tests did not agree. Figure conventions are the same as in Supplementary Figure 6, yet here the X-axis scale ranges between 0.2 and 0.9.

**
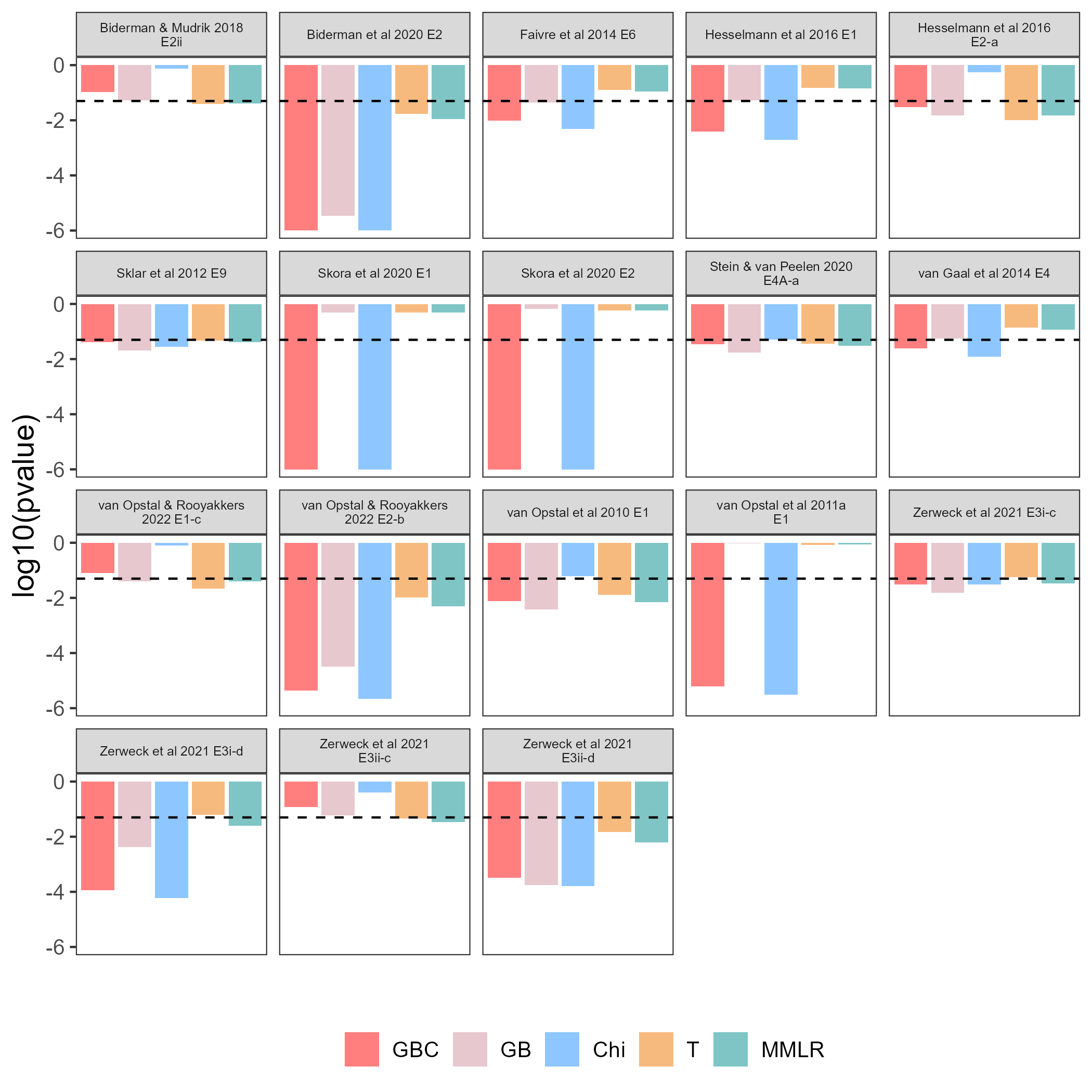
**

**Supplementary Figure 9:** P-values of the frequentist tests for tested effects on which the different awareness tests did not agree. The y-axis indicates log10(p-value). The colors show the different tests: GBC (red), GB (pink), Chi (blue), T (orange), and MMLR (green). The tested effect's name is written in the title, and their order is the same as in Figure 8 for ease of comparison. The horizontal black line denotes the significance threshold of all frequentist tests (p = .05), due to correction for multiple comparisons, the GBC p-values are twice the minimum p-value of its two constituent tests. P-values below ${10}^{-6}$ were modified to this value.


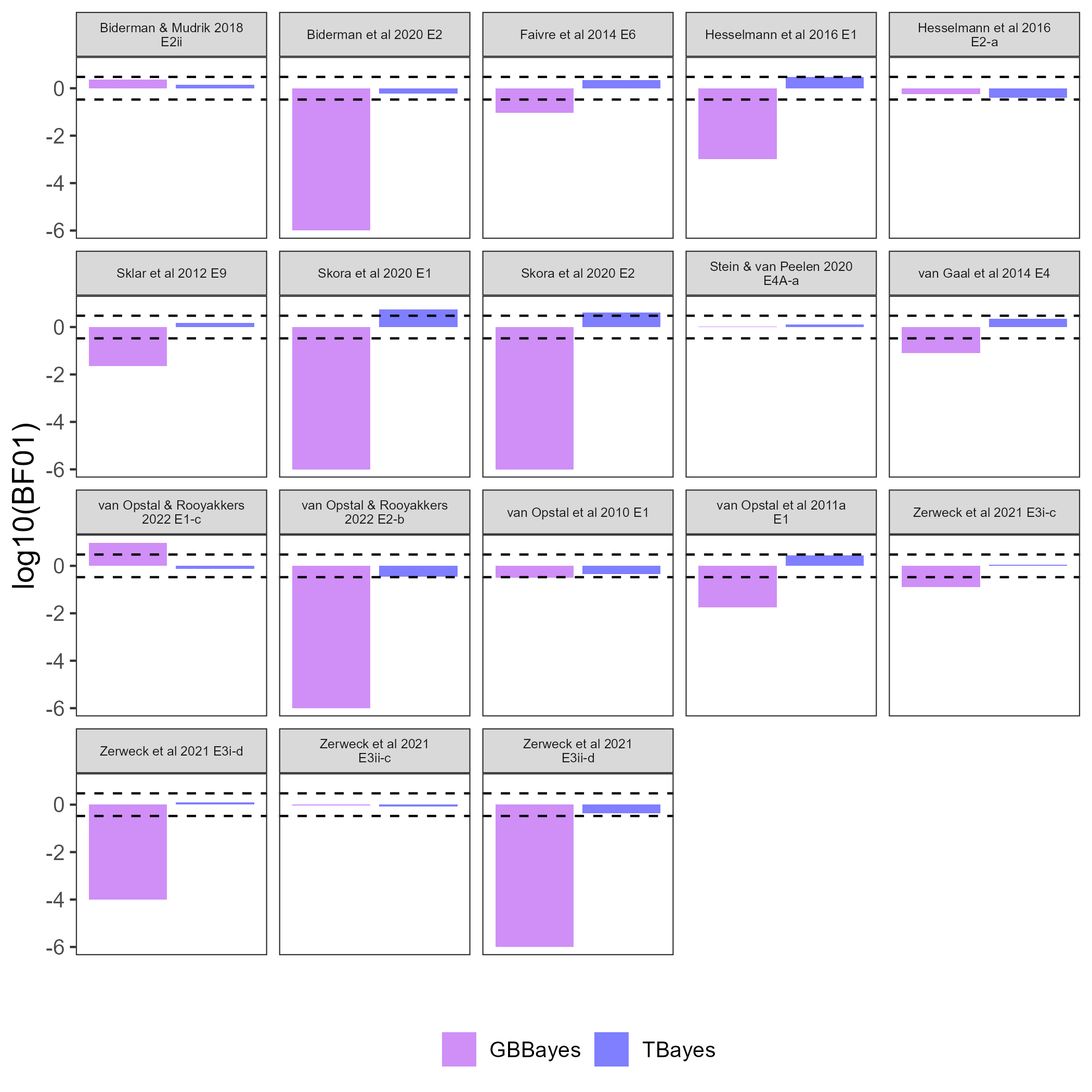


**Supplementary Figure 10:** BFs generated by the Bayesian tests for the tested effects that the different awareness tests did not agree on. The y-axis indicates log10($BF_{01}$). The color denotes the following tests: GB-Bayes (purple) and Bayesian t-test; TBayes (blue). The horizontal upper and lower black lines denote the threshold for moderate evidence for $H_{0}$ and $H_{1}$ of all Bayesian tests ($BF_{01}$= 3 and $BF_{01}$= 1/3), correspondingly (Evidence for $H_{0}$ is presented, so that the directionality for finding evidence for $H_{1}$ will be the same as in Figure 9). BFs below ${10}^{-6}$ were modified to this value. Figure conventions are the same as in Supplementary Figure 9.

**Chapter 6: Analysis of tested effects using the original participant exclusion criteria**

In the main analysis, we did not exclude participants due to their objective awareness scores, even if they had been excluded in the original experiment. This decision was driven by the ongoing concerns regarding post-hoc selection, potentially leading to falsely inferring unconscious effects (Shanks 2017). Yet because participant exclusion decreases the variability of awareness scores, this decision might have influenced the results of the tests that estimate the variability of awareness in the population based on the sample. Here, we repeated this analysis for frequentist tests focusing only on experiments that had a participant exclusion policy, which we now followed. To that end, we implemented two other frequentist tests, in which the same rationale of GB and Chi-squared tests were used, yet while adding participant exclusion to the null hypothesis. The first, **Resampling with Criterion (RC) test** follows the GB test, and the second, **Absolute Value Resampling with Criterion (AVRC) test** follows the Chi-squared test. Similarly to the analysis described in the main manuscript, we examined the results of a test combining both (setting the p-value of the combined test to be twice the minimal p-value between the RC and AVRC tests). In these tests, the group level accuracy is tested against chance by comparing the estimated awareness to a resampled null distribution, where awareness scores for each participant are randomly drawn from the predefined binomial distribution ($p_{i}=0.5$, $n_{i}=$ number of trials for participant i), to mimic a situation that all participants are unaware. Then, participants are excluded according to the same criterion used in the experimental dataset (which was either defined using a binomial test or a fixed threshold). In addition, when the exclusion of participants has led to included samples of less than 10 participants, we excluded it from the analysis to avoid extremely small samples. Thus, the main difference between the RC test and the GB test is that the former uses resampling and accordingly does not assume normality. For the null distribution, in this analysis we compared the performance of the combined test to that of the t-test and MMLR (for completeness, we also show the results for RC and AVRC separately). The AVRC is identical to the RC, but transforms awareness scores in the following way:

$X_{i}' = 0.5 + |0.5 - X_{i}|$ (thus, all scores are above chance).

In 22 out of the 24 tested effects, all tests agreed. In one tested effect, the combined RC or AVRC test did not detect an above-chance performance while the t-test and MMLR did, and in another, it was the MMLR that did not detect above chance-performance. Thus, these results corroborate the conclusion of the main analysis reported in the manuscript.

**
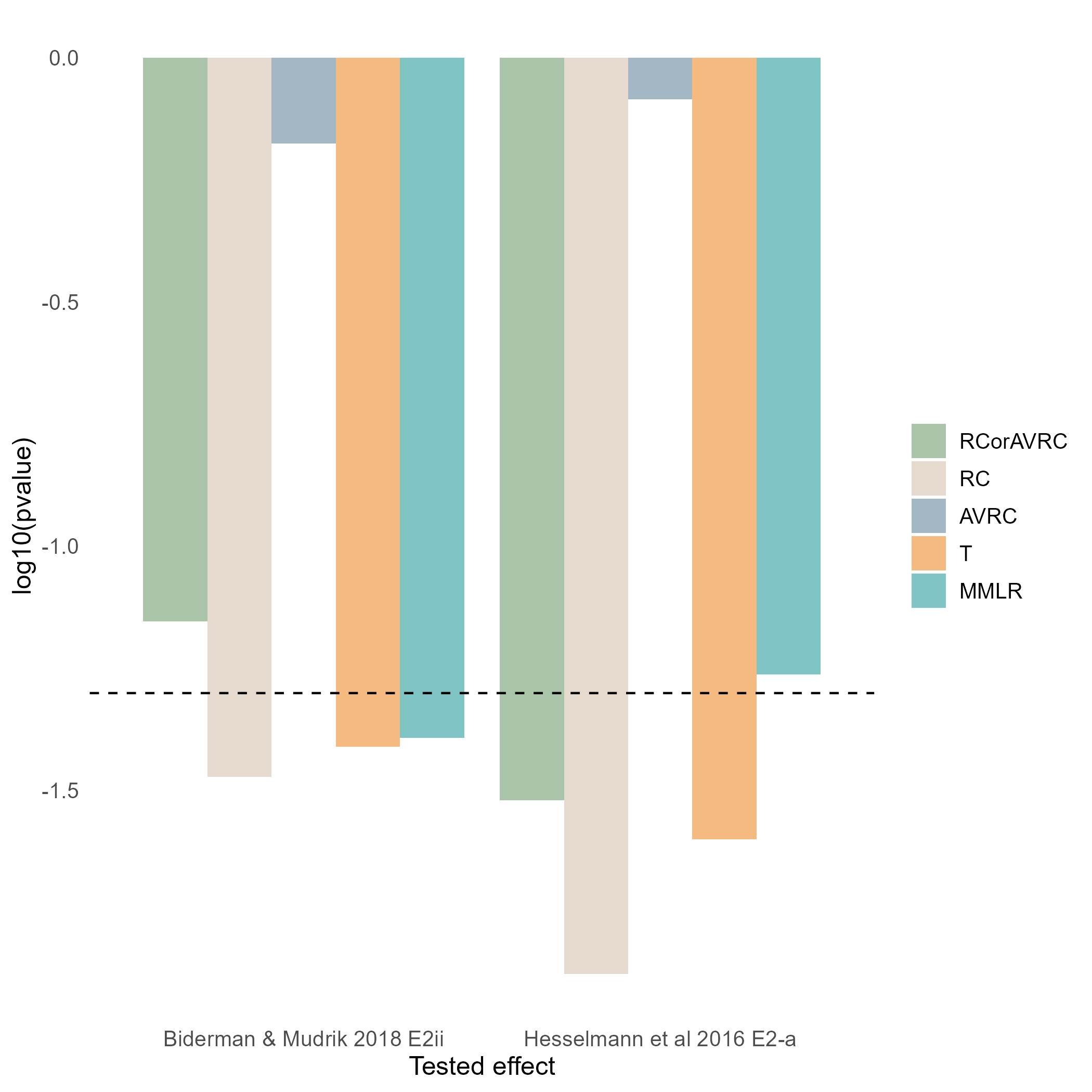
**

**Supplementary Figure 11:** Datasets re-analysis after post-hoc selection of participants: only tested effects in which there was a disagreement between the 3 tests (RCorAVRC test, t-test, and MMLR) on the significance of the group are presented (N = 2; in the remaining 22 tested effects that have a criterion for participant exclusion all tests found significant or nonsignificant results). The horizontal black line denotes the significance threshold for all frequentist tests (p = .05). Due to correction for multiple comparisons, the RCorAVRC p-values are twice the minimum p-value of its two constituent tests. Each color depicts the p-value of a different test: RCorAVRC (light green), RC (khaki), AVRC (gray), T (orange), MMLR (green). Conventions are the same as in Figure 3.

**Chapter 7: Characterization of studies included in datasets re-analysis**

All tested effects were collected from experiments examining unconscious processing and included an objective measure of awareness. The table below provides meta-data on these reported studies, and additional comments that came up in the analysis process.

**Supplementary Table 1:** Summary of reported studies

| **Label** | **DOI** | **Awareness measures** | **The process of interest** | **Number of tested effects** | **Average Sample size** | **Average number of trials in the objective awareness measure** |
| --- | --- | --- | --- | --- | --- | --- |
| Benthien & Hesselmann 2021 | <https://doi.org/10.5709%2Facp-0312-3> | Objective and Subjective | Location uncertainty and numerical processing | 2 | 26 | 46 |
| Skora et al 2020^^[[3]](#footnote-3)^^ | <https://doi.org/10.1016/j.cognition.2020.104546> | Objective and Subjective | Reinforcement learning | 2 | 27 | 128 |
| Zerweck et al 2021^^[[4]](#footnote-4)^^ | <https://doi.org/10.1177/014662168300700301> | Objective | Numerical processing | 25 | 15 | 104 |
| Stein & Van Peelen 2020 | <https://doi.org/10.1038/s41562-020-01004-5> | Objective and Subjective (exp 3), Objective (exp 4) | Exp 3 - Face orientation processing  Exp 4 - Object processing | 13 | 65 | 174 |
| Biderman et al 2020^^[[5]](#footnote-5)^^ | <https://doi.org/10.1177/0956797620915887> | Objective and Subjective | Context processing | 5 | 32 | 86 |
| Karpinski et al 2018^^[[6]](#footnote-6)^^ | <https://doi.org/10.1002/ejsp.2390> | Objective and Subjective | Arithmetics | 1 | 114 | 64 |
| Hesselmann et al 2016^^[[7]](#footnote-7)^^ | <https://doi.org/10.1167/16.3.17> | Objective and Subjective | Object and shape processing | 3 | 28 | 67 |
| Biderman & Mudrik 2018 | <https://doi.org/10.1177/0956797617735745> | Objective and Subjective | Object-scene congruency | 6 | 48 | 192 |
| van Gaal et al 2014 | <https://doi.org/10.1098/rstb.2013.0212> | Objective | Words integration | 1 | 16 | 40 |
| Sklar et al 2012^^[[8]](#footnote-8)^^ | <https://doi.org/10.1073/pnas.1211645109> | Objective and Subjective | Arithmetics | 3 | 44 | 59 |
| van Opstal et al 2010 | <https://doi.org/10.1016/j.concog.2010.05.002> | Objective and Subjective | Task application | 3 | 20 | 144 |
| van Opstal et al 2011a | <https://doi.org/10.1016/j.cognition.2011.03.005> | Objective and Subjective | Semantic processing | 2 | 23 | 100 |
| van Opstal et al 2011b | https://doi.org/10.1016/j.concog.2011.09.004 | Objective and Subjective | Context processing | 1 | 19 | 144 |
| van Opstal & Rooyakkers 2022 | <https://doi.org/10.1016/j.cognition.2022.105113> | Objective | Symbol integration | 6 | 25 | 160 |
| Faivre et al 2014^^[[9]](#footnote-9)^^ | <https://doi.org/10.1177/0956797614547916> | Objective (all exp) Objective and subjective (exp 3) | Multisensory integration | 6 | 22 | 101 |

**References**

Donhauser, P. W., Florin, E., & Baillet, S. (2018). Imaging of neural oscillations with embedded inferential and group prevalence statistics. *PLoS Computational Biology*, *14*(2), e1005990. https://doi.org/10.1371/journal.pcbi.1005990

1. The false positive rate of the individual tests was calculated in each condition according to the number of simulated trials as the expected false-positive rate of binomial tests (which is lower than the $\alpha$ level used in these tests due to the discreteness of binomial distributions). Furthermore, we used a rejection region including both values below the 2.5% of the cumulative binomial distribution and the value above the lowest value that keeps the overall rejection region up to 5%. In other words, our rejection zone was not necessarily symmetrical, with a slight preference for rejecting above-chance performance. [↑](#footnote-ref-1)
2. Note that since the true $p$ of each participant is limited between (0,1), the standard deviations of the different $p_{i}$ in the aware group are also limited. Accordingly, we set the prior on $\sigma$ to distribute uniformly between 0 and the maximal possible standard deviation of the aware participants, which is 0.5. [↑](#footnote-ref-2)
3. Skora 2020: in our analysis, trial exclusion diverged from that used in the paper. We defined aware trials as all confident trials, independent of the performance. In the paper, aware trials were defined as confident and correct trials. [↑](#footnote-ref-3)
4. Zerweck 2021: while our reanalysis was based on the code provided with the paper, it yielded some numerical inconsistencies with the results reported in the paper. [↑](#footnote-ref-4)
5. Biderman 2020: Experiment 4b: the exclusion criteria was performance > 0.65 in at least one of the objective awareness tests. Nonetheless, one participant had such performance in the categorical task and yet was not excluded for some reason. Here, we did exclude that participant. [↑](#footnote-ref-5)
6. Karpinski Briggs Yale 2018: We included one participant who was originally excluded due to a technical error in the data file. [↑](#footnote-ref-6)
7. Hesselmann 2016: a minor inconsistency was found in the average awareness scores for Experiment 1. [↑](#footnote-ref-7)
8. Sklar 2012: 1) In experiments 6 and 7, we estimated the number of correct trials (as it was not reported in the original manuscript) based on the reported awareness scores. 2). Some inconsistencies in the p-values were found. None but one changed the conclusion of the analyses in that one (Experiment 7) we found evidence for above chance performance. 3) In the sample of Experiment 7, we included 28 participants who were originally excluded for being objectively aware. However, the original manuscript reports 30 participants who were objectively aware. The reason two participants were not included in our analysis is that they were also subjectively aware. 3) In Experiment 9, we only included four objectively aware participants, since two of them were also subjectively aware and one had ceiling performance in the incongruent condition, which was defined as an exclusion criterion in the original work. [↑](#footnote-ref-8)
9. Faivre 2014: In Experiment 6 (which was only reported in the supplementary materials in the original publication), the data for one participant was missing. In addition, the reported average performance (0.522) seems to have been calculated including one participant who was supposed to be excluded based on the predefined criterion. [↑](#footnote-ref-9)
