# Supplementary figures and images for: Increasing power for detecting awareness: a new approach to test group level objective performance
Source: Neurosci Conscious. 2026 Jul 28;2026(1):niag039. doi: 10.1093/nc/niag039 (PMC13411290; doi:10.1093/nc/niag039)

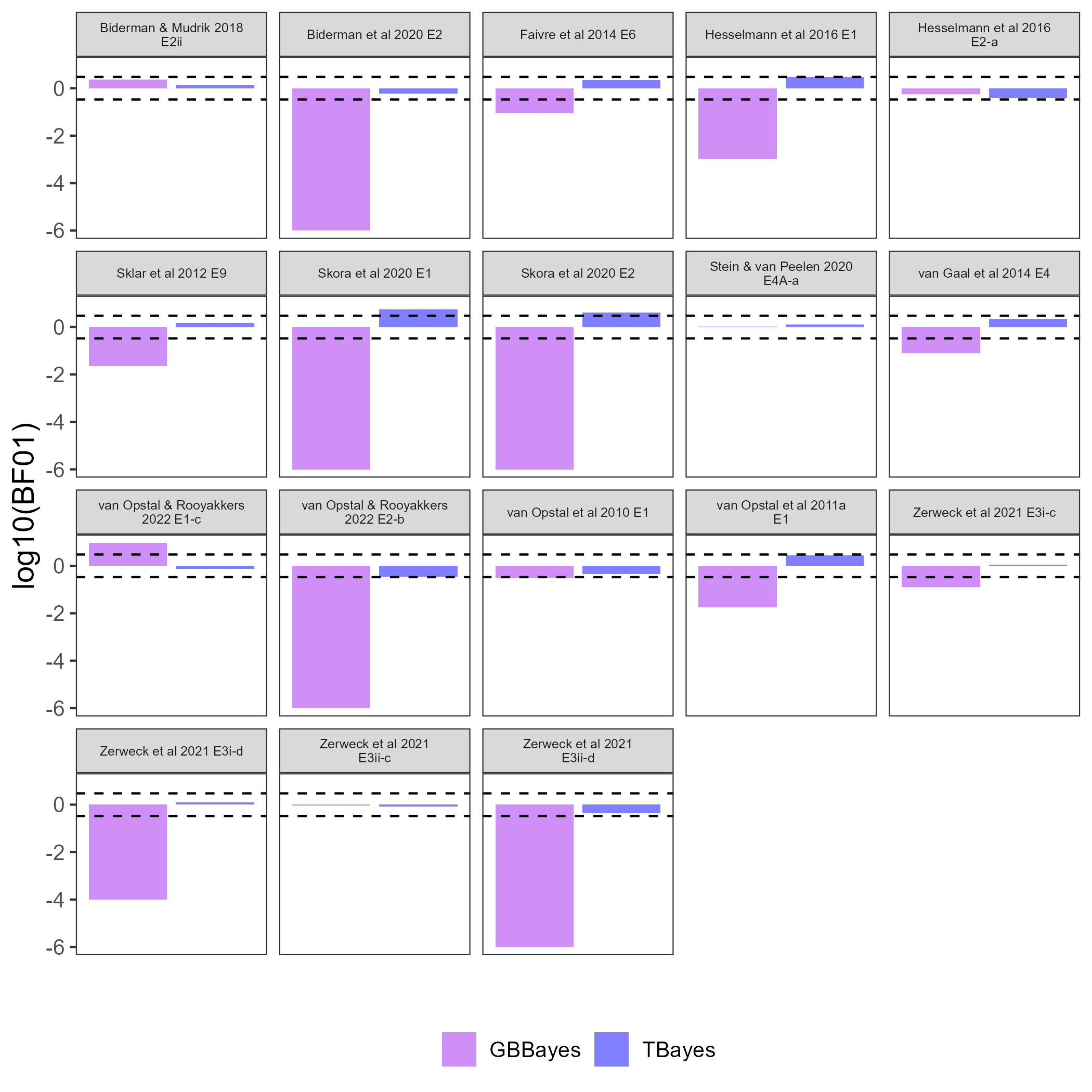

Supplement: Supplementary_material_niag039 [file supplementary_material_niag039.zip › Supplementary_Figure_10_niag039.png]

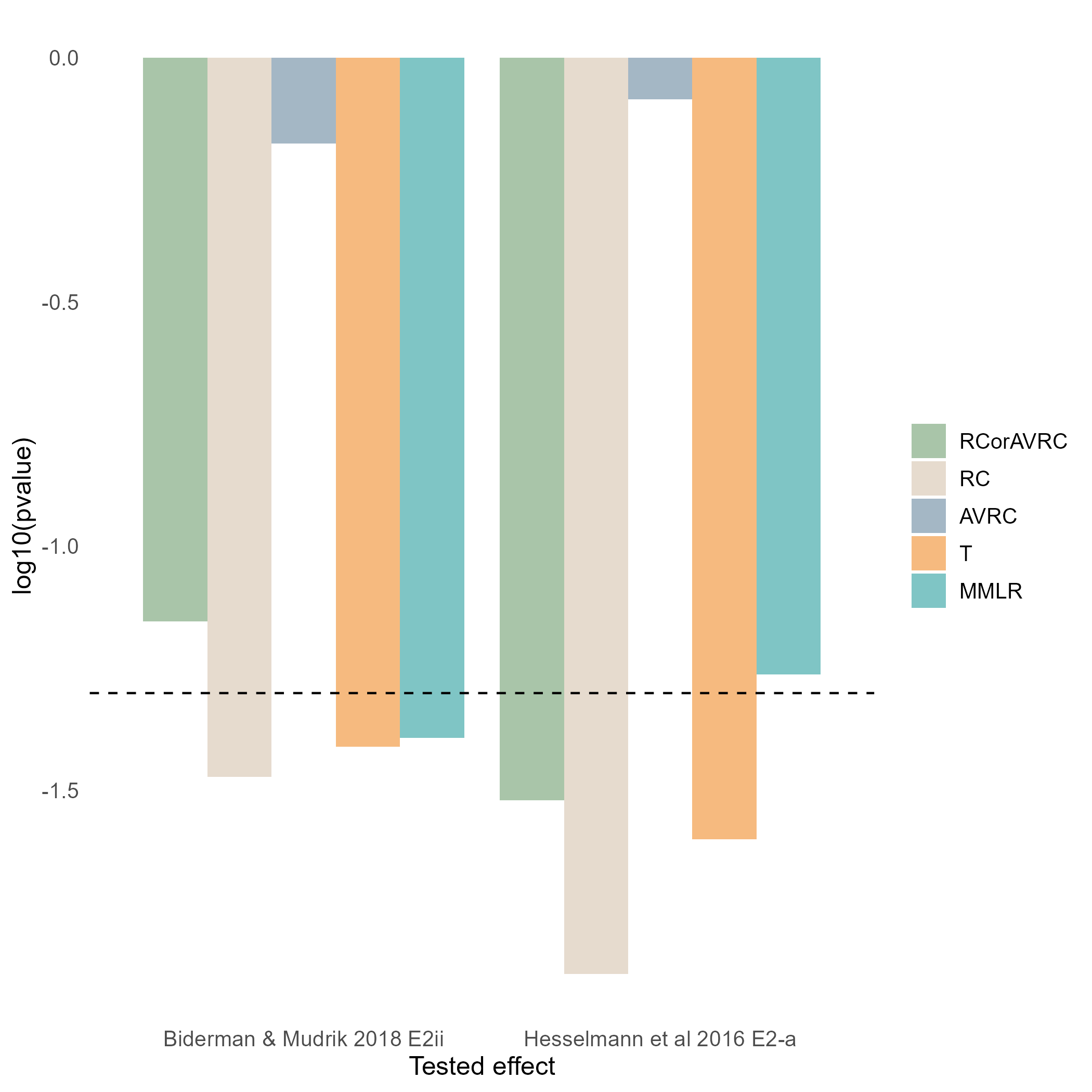

Supplement: Supplementary_material_niag039 [file supplementary_material_niag039.zip › Supplementary_Figure_11.png]

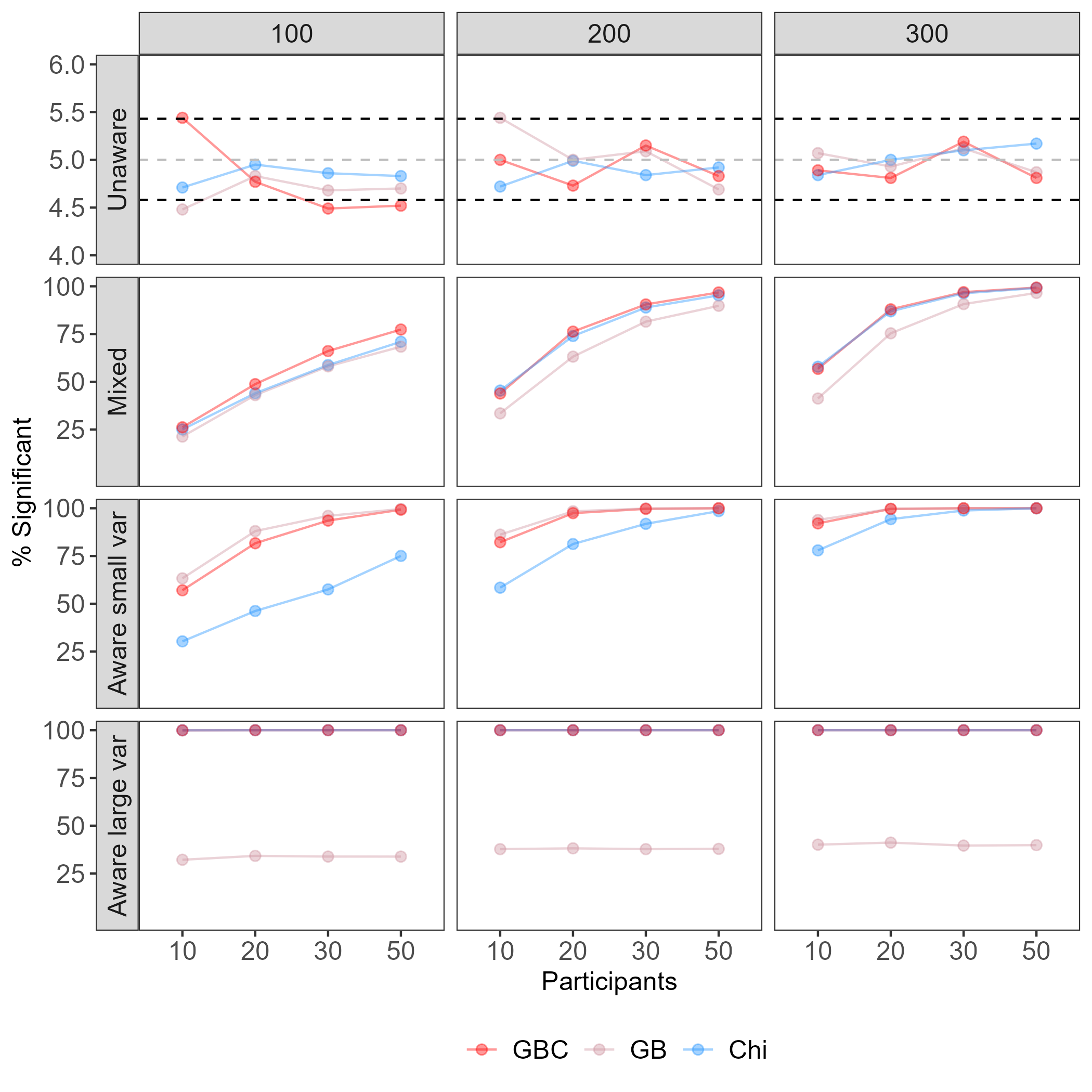

Supplement: Supplementary_material_niag039 [file supplementary_material_niag039.zip › Supplementary_Figure_1_niag039.png]

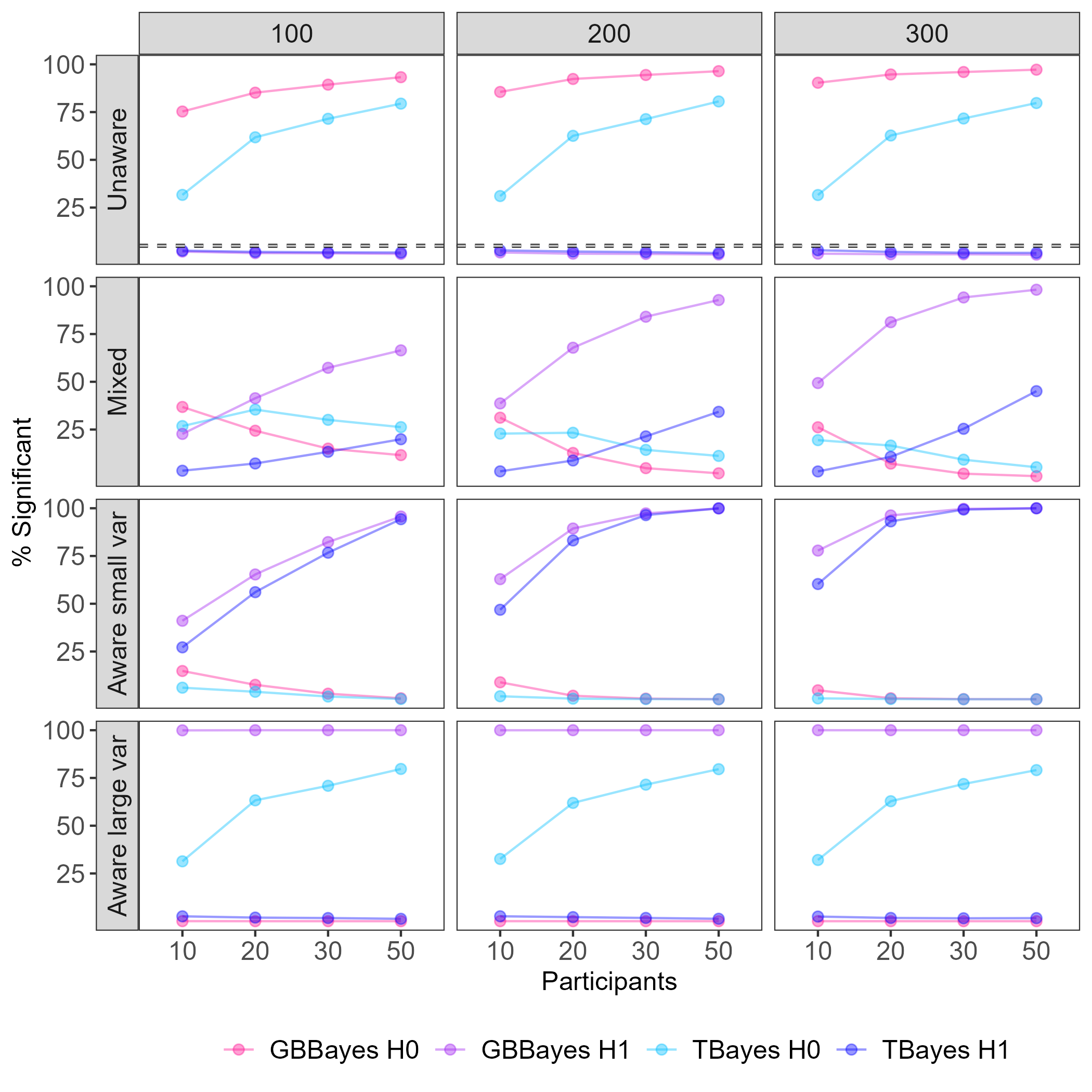

Supplement: Supplementary_material_niag039 [file supplementary_material_niag039.zip › Supplementary_Figure_2_niag039.png]

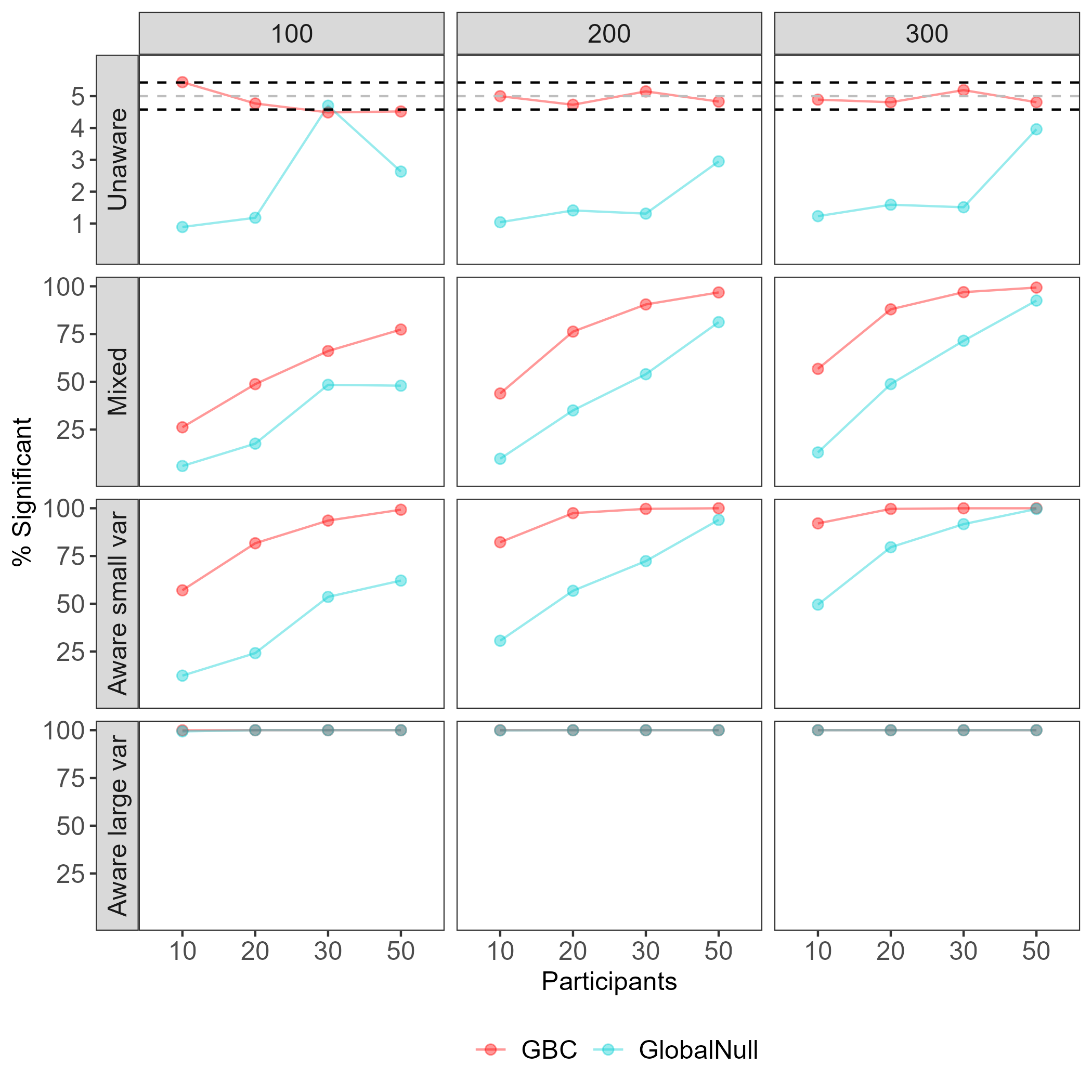

Supplement: Supplementary_material_niag039 [file supplementary_material_niag039.zip › Supplementary_Figure_3_a_niag039.png]

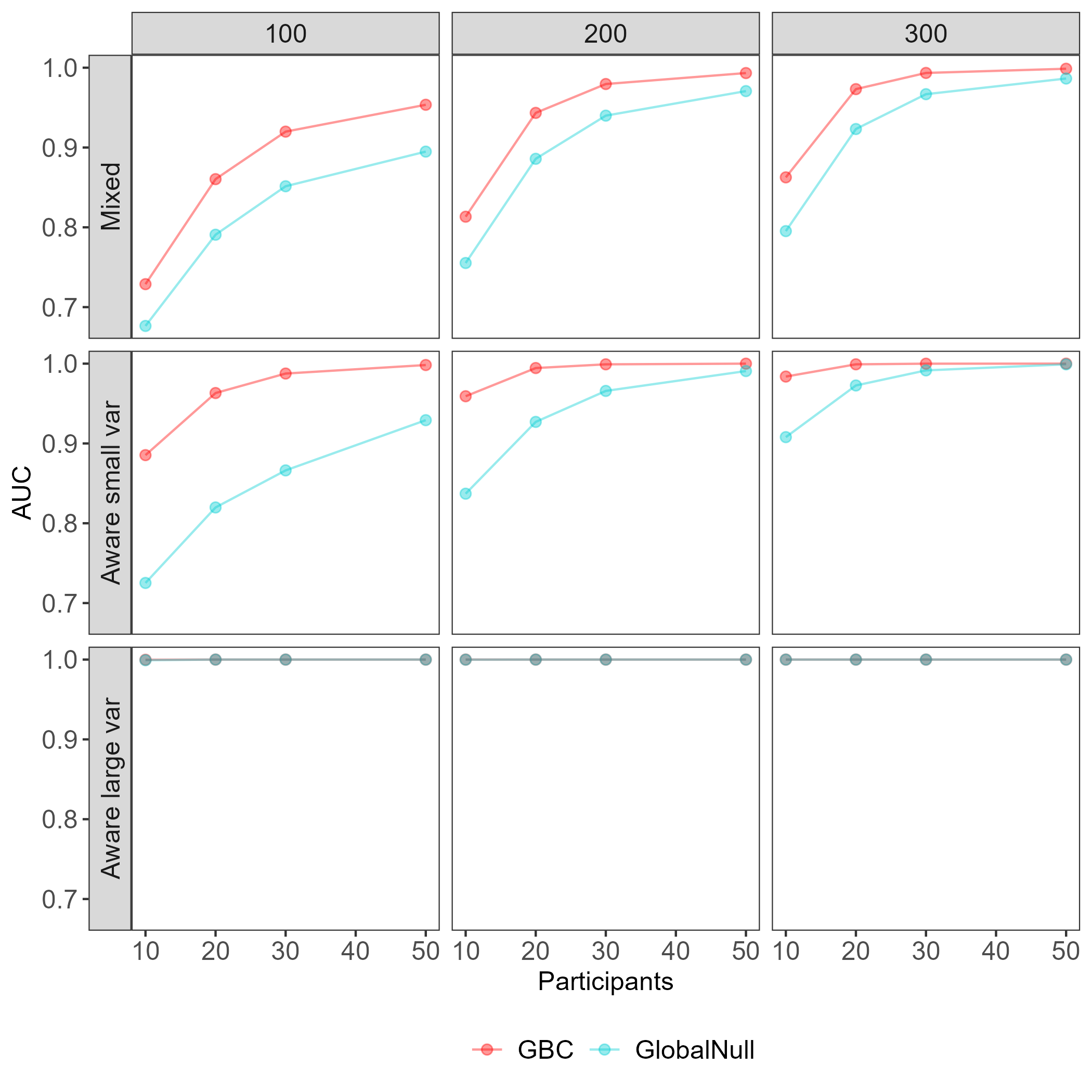

Supplement: Supplementary_material_niag039 [file supplementary_material_niag039.zip › Supplementary_Figure_3_b_niag039.png]

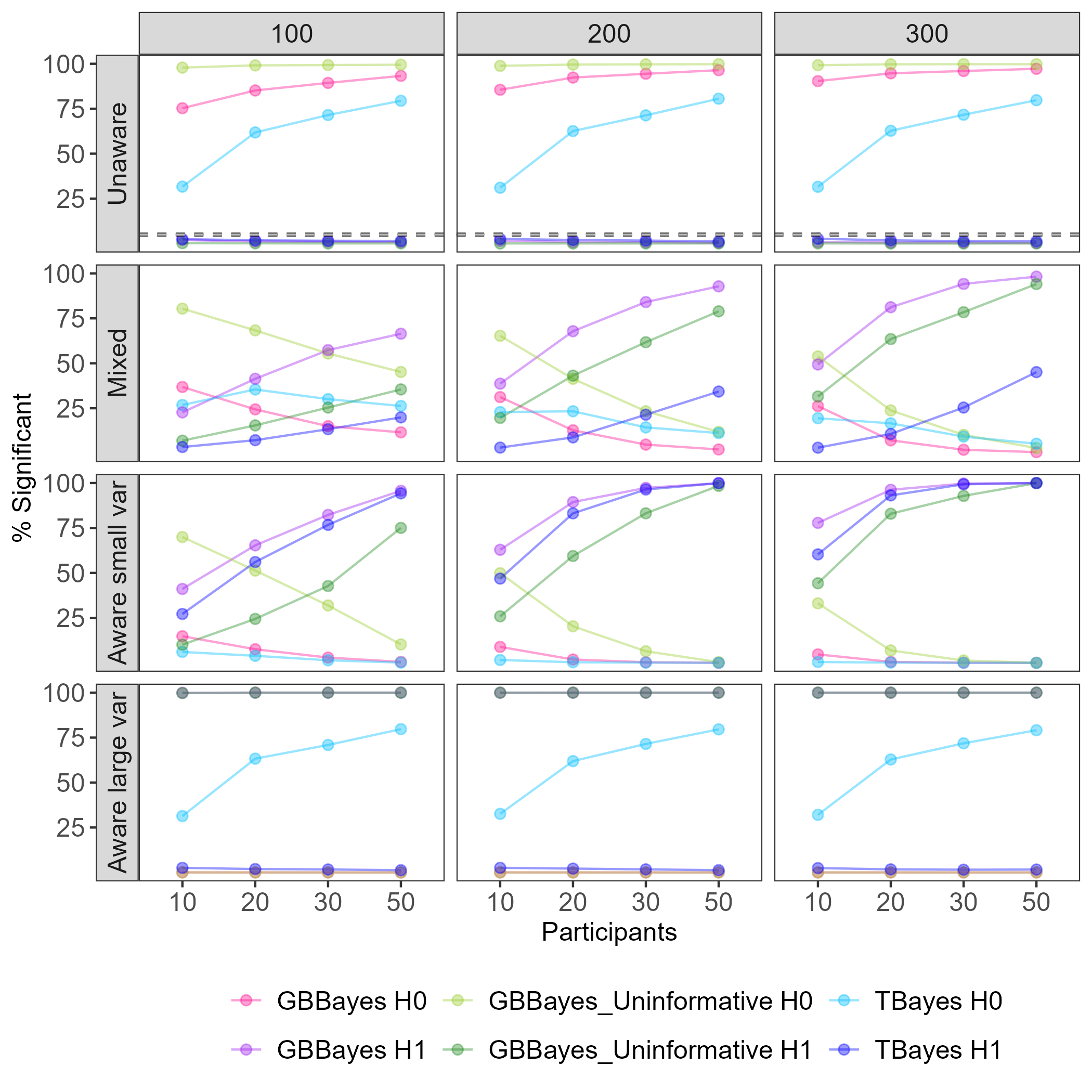

Supplement: Supplementary_material_niag039 [file supplementary_material_niag039.zip › Supplementary_Figure_4_a_niag039.png]

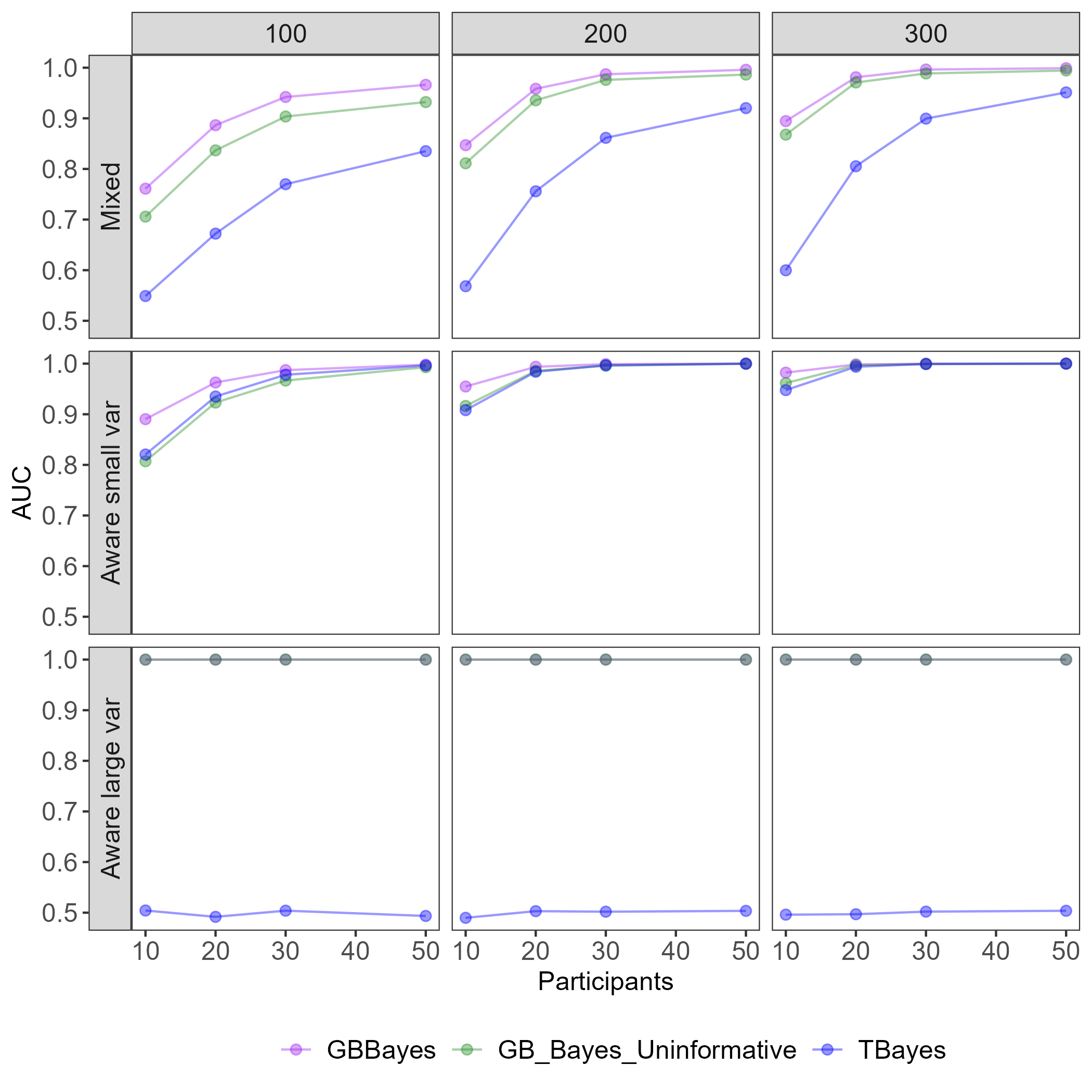

Supplement: Supplementary_material_niag039 [file supplementary_material_niag039.zip › Supplementary_Figure_4_b_niag039.png]

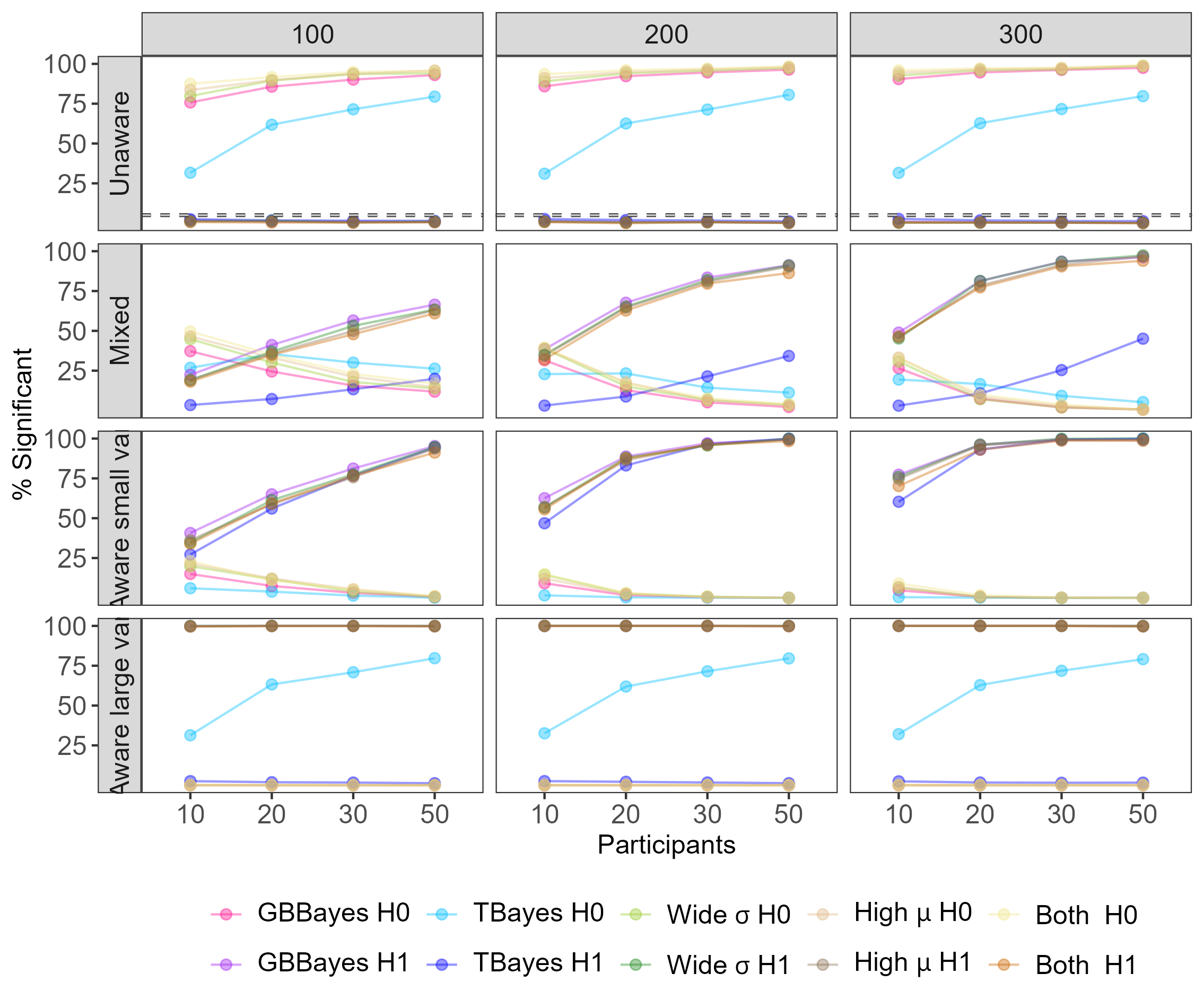

Supplement: Supplementary_material_niag039 [file supplementary_material_niag039.zip › Supplementary_Figure_5_a_niag039.png]

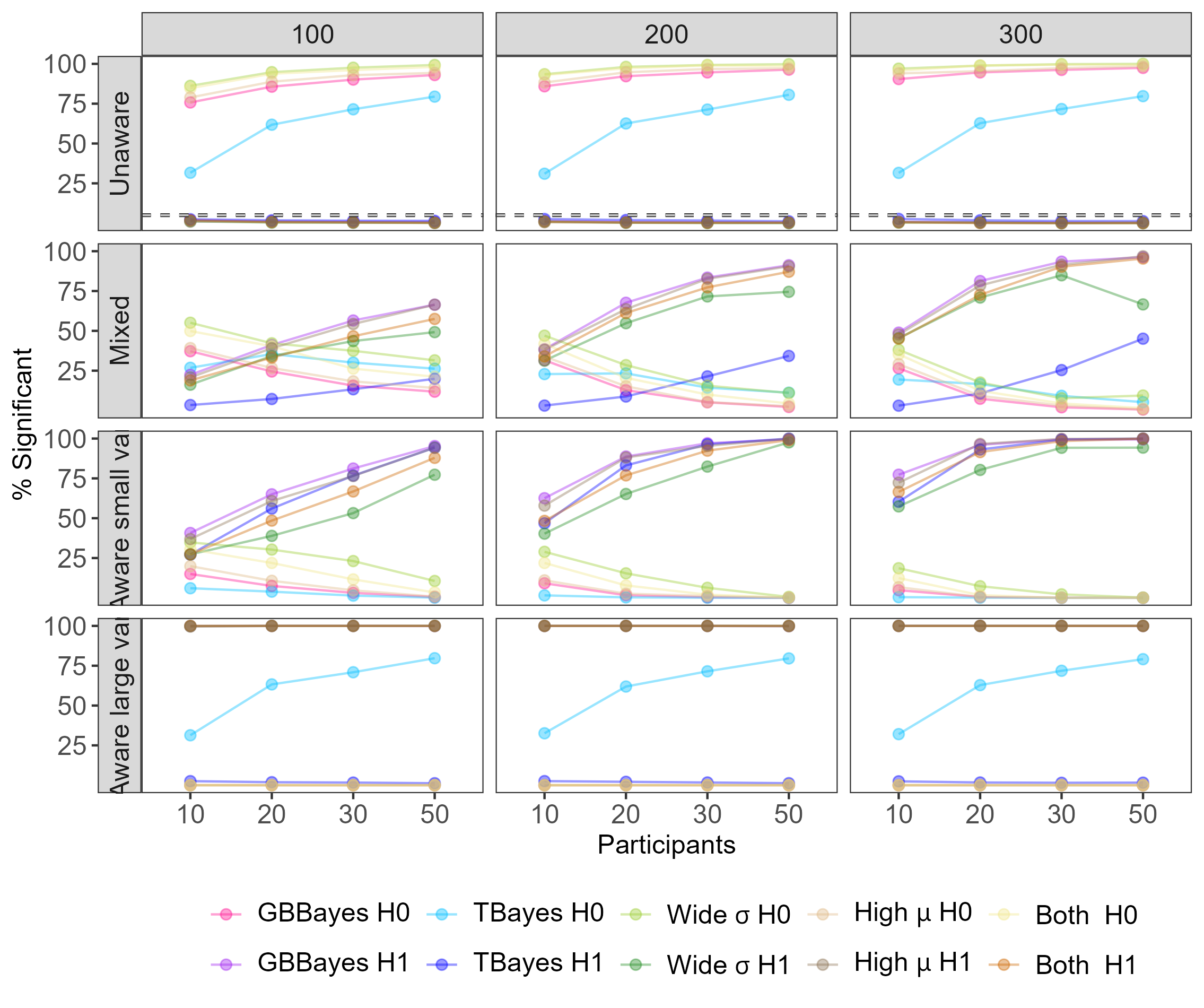

Supplement: Supplementary_material_niag039 [file supplementary_material_niag039.zip › Supplementary_Figure_5_b_niag039.png]

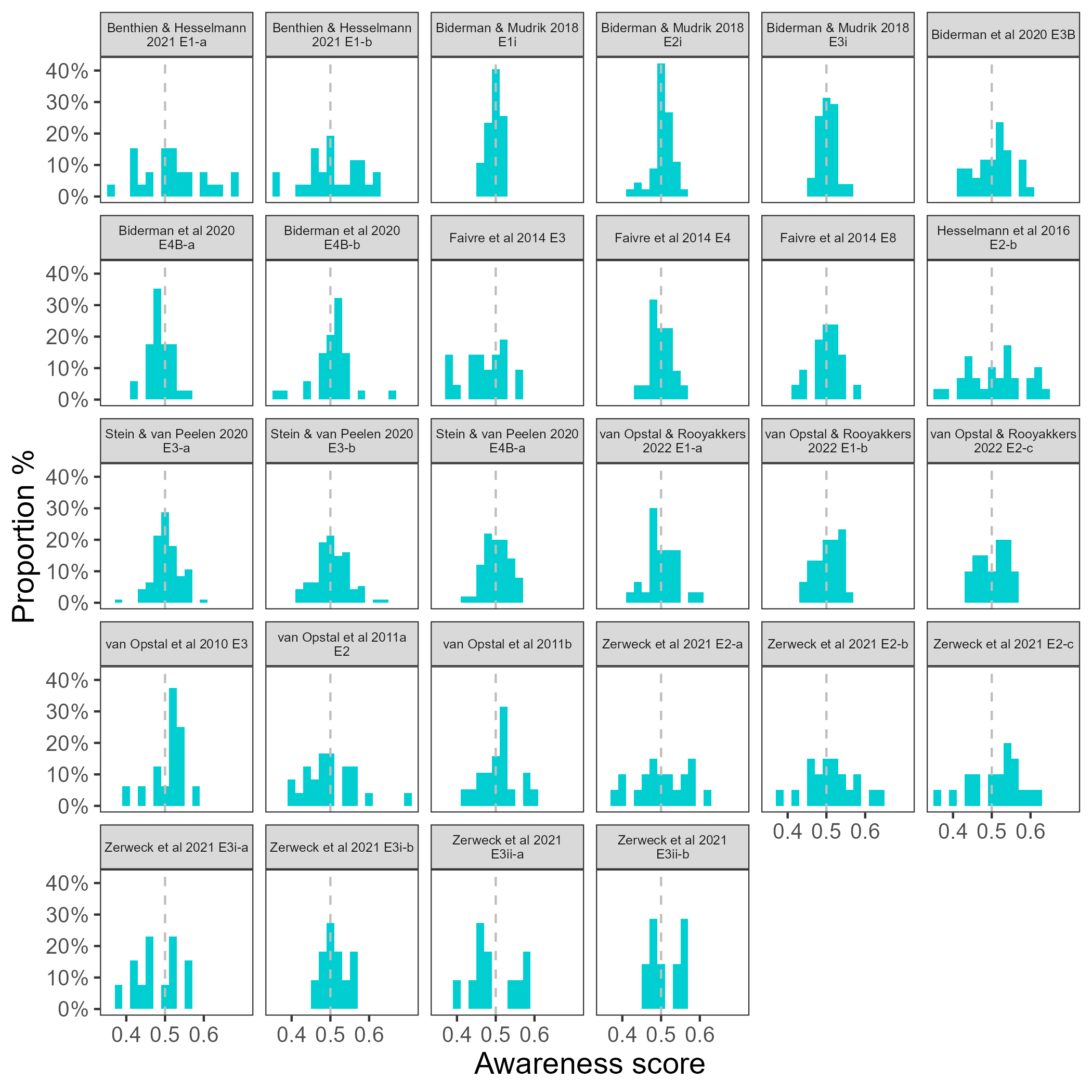

Supplement: Supplementary_material_niag039 [file supplementary_material_niag039.zip › Supplementary_Figure_6_niag039.png]

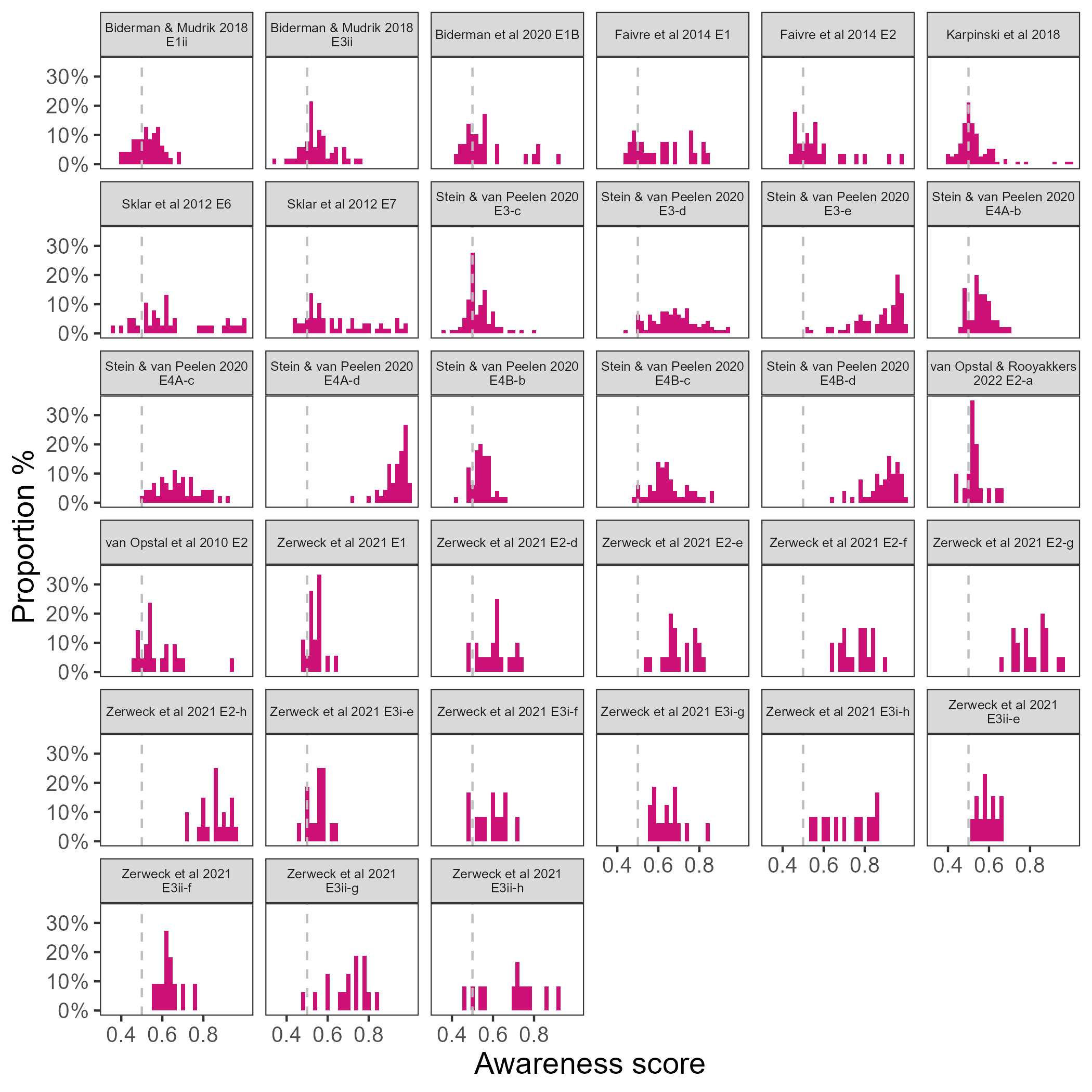

Supplement: Supplementary_material_niag039 [file supplementary_material_niag039.zip › Supplementary_Figure_7_niag039.png]

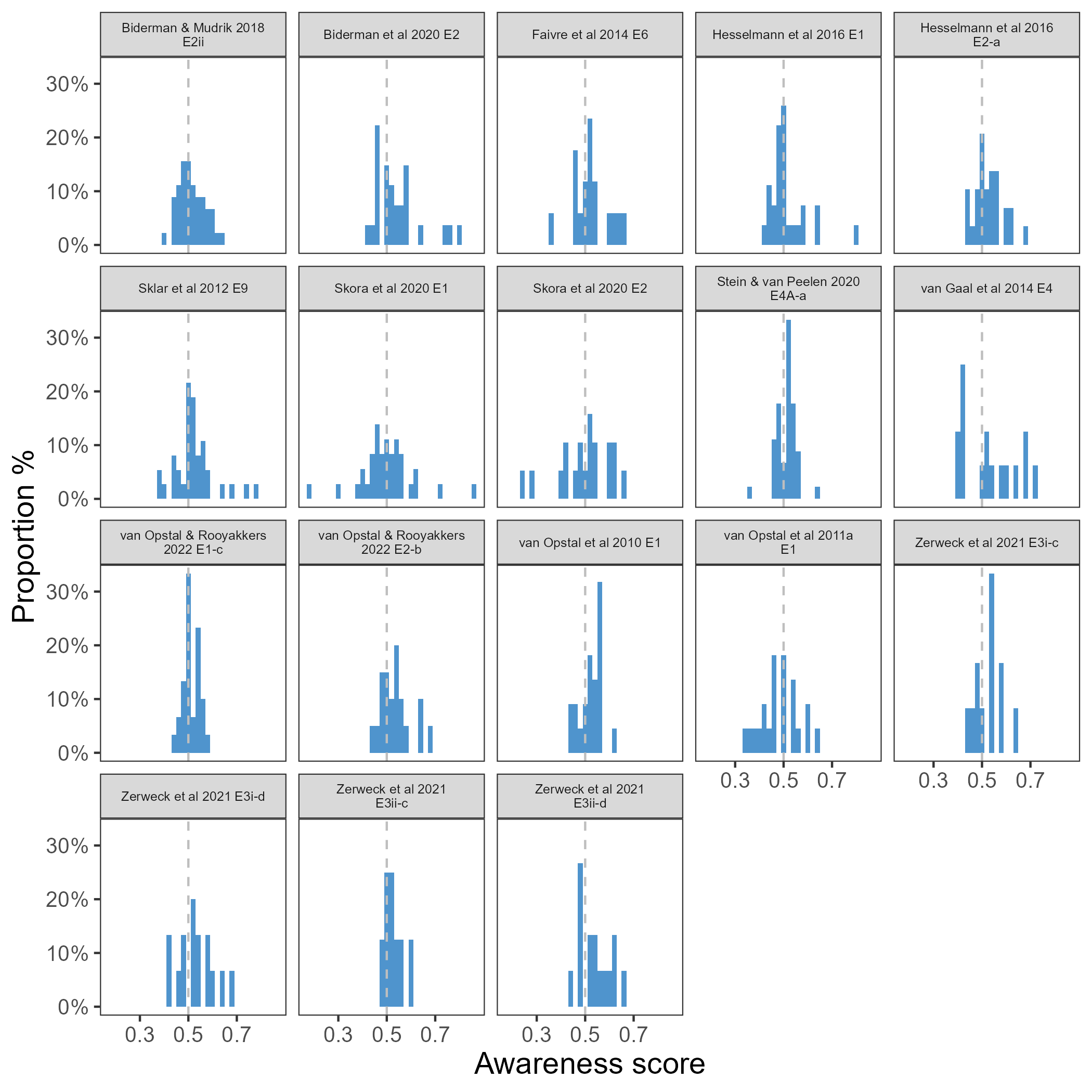

Supplement: Supplementary_material_niag039 [file supplementary_material_niag039.zip › Supplementary_Figure_8_niag039.png]

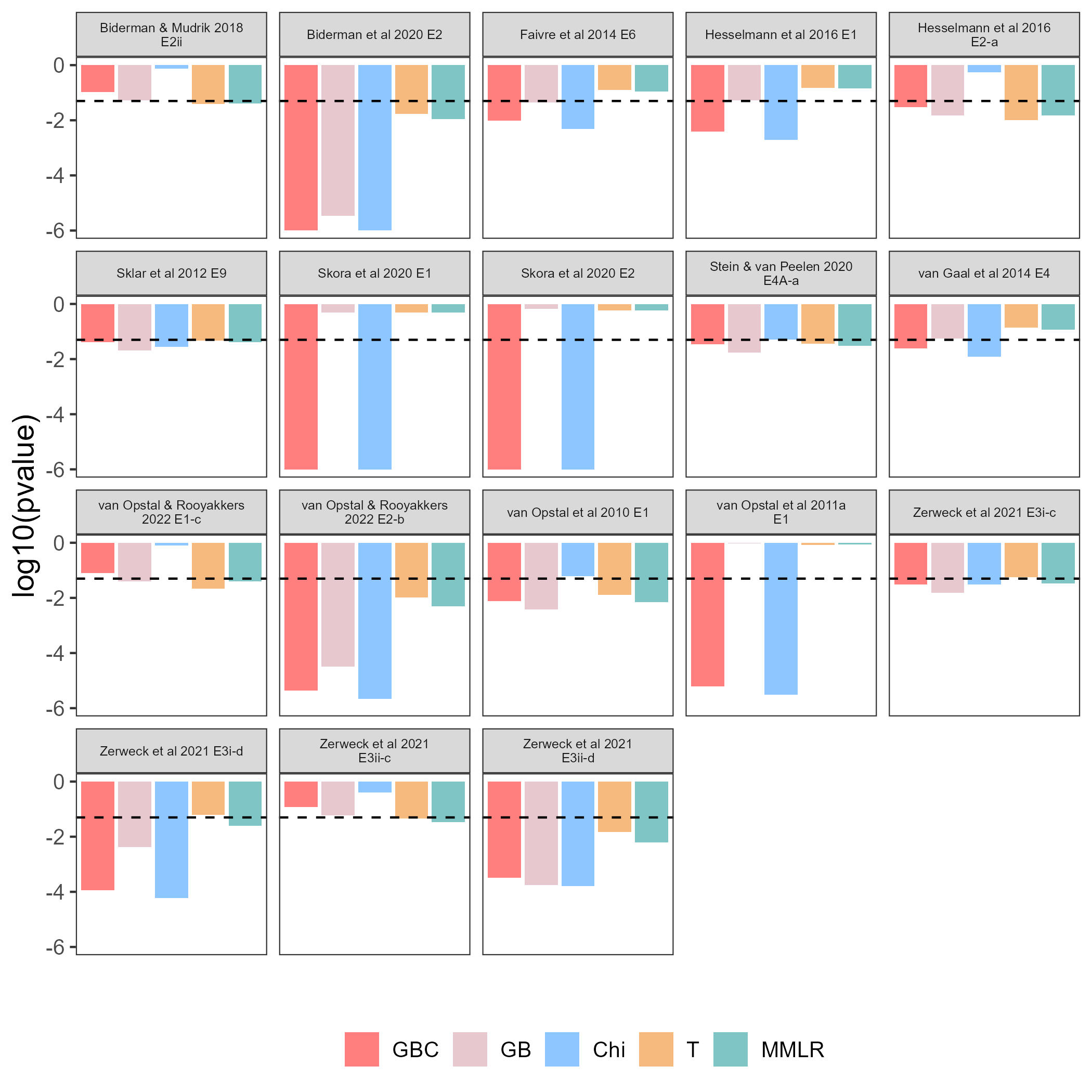

Supplement: Supplementary_material_niag039 [file supplementary_material_niag039.zip › Supplementary_Figure_9_niag039.png]
